# Hypoxic Upregulation of IER2 Increases Paracrine GMFG Signaling of Endoplasmic Reticulum Stress‐CAF to Promote Chordoma Progression via Targeting ITGB1

**DOI:** 10.1002/advs.202405421

**Published:** 2024-08-29

**Authors:** Tao‐Lan Zhang, Bo‐Wen Zheng, Chao Xia, Peng‐Fei Wu, Bo‐Yv Zheng, Ling‐Xiang Jiang, Jing Li, Guo‐Hua Lv, Hong Zhou, Wei Huang, Ming‐Xiang Zou

**Affiliations:** ^1^ Department of Pharmacy The First Affiliated Hospital Hengyang Medical School University of South China Hengyang 421001 China; ^2^ Musculoskeletal Tumor Center Peking University People's Hospital Peking University Beijing 100044 China; ^3^ Department of Spine Surgery The First Affiliated Hospital Hengyang Medical School University of South China Hengyang 421001 China; ^4^ Department of Genetics and Endocrinology National Children's Medical Center for South Central Region Guangzhou Women and Children's Medical Center Guangzhou Medical University Guangzhou Guangdong 510623 China; ^5^ Department of Orthopedics Surgery General Hospital of the Central Theater Command Wuhan 430061 China; ^6^ Department of Radiation Oncology Melvin and Bren Simon Comprehensive Cancer Center Indiana University School of Medicine Indianapolis IN 46202 USA; ^7^ Department of Spine Surgery The Second Xiangya Hospital Central South University Changsha 410011 China; ^8^ Department of Radiology The First Affiliated Hospital Hengyang Medical School University of South China Hengyang 421001 China; ^9^ The First Affiliated Hospital Health Management Center Hengyang Medical School University of South China Hengyang 421001 China

**Keywords:** cancer‐associated fibroblasts, chordoma progression, endoplasmic reticulum stress, hypoxia, IER2/GMFG/ITGB1 axis, paracrine

## Abstract

Currently, the oncogenic mechanism of endoplasmic reticulum stress‐CAF (ERS‐CAF) subpopulation in chordoma remains unknown. Here, single‐cell RNA sequencing, spatial transcriptomics, GeoMx Digital Spatial Profiler, data‐independent acquisition proteomics, bulk RNA‐seq, and multiplexed quantitative immunofluorescence are used to unveil the precise molecular mechanism of how ERS‐CAF affected chordoma progression. Results show that hypoxic microenvironment reprograms CAFs into ERS‐CAF subtype. Mechanistically, this occurrs via hypoxia‐mediated transcriptional upregulation of IER2. Overexpression of IER2 in CAFs promotes chordoma progression, which can be impeded by IER2 knockdown or use of ERS inhibitors. IER2 also induces expression of ERS‐CAF marker genes and results in production of a pro‐tumorigenic paracrine GMFG signaling, which exert its biological function via directly binding to ITGB1 on tumor cells. ITGB1 inhibition attenuates tumor malignant progression, which can be partially reversed by exogenous GMFG intervention. Further analyses reveal a positive correlation between ITGB1^high^ tumor cell counts and SPP1^+^ macrophage density, as well as the spatial proximity of these two cell types. Clinically, a significant correlation of high IER2/ITGB1 expression with tumor aggressive phenotype and poor patient survival is observed. Collectively, the findings suggest that ERS‐CAF regulates SPP1^+^ macrophage to aggravate chordoma progression via the IER2/GMFG/ITGB1 axis, which may be targeted therapeutically in future.

## Graphical abstract

The hypoxic chordoma microenvironment induces upregulation of IER2 in CAF to stimulate its ERS effect and then release GMFG cytokine. GMFG secreted by ERS‐CAF reshapes the immune microenvironment possibly via recruiting SPP1^+^ macrophages to promote chordoma progression by binding to ITGB1 on tumor cells. This study elucidates the specific molecular mechanism by which ERS‐CAF regulated chordoma progression.

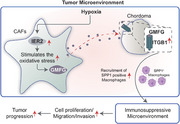

## 1 Introduction

Chordoma, a rare mesenchymal malignant tumor, is considered to arise from residual notochord tissue during embryonic development. Predominantly located in axial bones, especially the skull base and sacrococcygeal region,^[^ ^1^ ^]^ chordoma exhibits slow and insidious growth nature, hampering early detection. Typically, diagnosis occurs late, by which point the tumor has infiltrated surrounding tissues with indistinct margins.^[^ ^2^ ^]^ Due to its insensitivity to conventional radiotherapy and chemotherapy, the ideal treatment for chordoma at present involves maximal tumor resection during the initial surgery combined with adjuvant radiotherapy.^[^ ^3^, ^4^ ^]^ However, the intricate anatomical proximity to neurovascular structures complicates achieving complete surgical resection. This presents a medical dilemma, balancing preservation of organ and neurological function against disease eradication.^[^ ^4^ ^]^ The prognosis of chordoma is bleak with a postoperative recurrence rate of up to 50%, and many patients eventually die due to the recurrent tumors and associated complications.^[^ ^5^, ^6^, ^7^ ^]^ In‐depth research into chordoma's molecular mechanisms and thus developing innovative therapeutic approaches are imperative to improve patient survival.

In our previous study, a distinct subset of cancer‐associated fibroblasts (CAFs) linked to endoplasmic reticulum stress (ERS) was identified within the chordoma microenvironment, namely “ERS‐CAFs”.^[^ ^8^ ^]^ This ERS‐CAF subtype governed multiple signaling pathways relevant to chordoma development and displayed elevated expression of hypoxia‐related genes.^[^ ^8^ ^]^ Hypoxia, a hallmark of tumorigenesis, can trigger ERS in cells by disrupting protein folding due to oxygen homeostasis imbalance, causing accumulation of misfolded proteins in the endoplasmic reticulum lumen.^[^ ^9^, ^10^, ^11^ ^]^ Conversely, altered phosphorylation of ERS‐related proteins (such as eIF2α, PERK, CHOP, and ATF4) escalates intracellular reactive oxygen species (ROS) levels, which can disrupt oxidative balance and thus promote disease progression.^[^ ^12^ ^]^ Existing evidence underscores ERS's role in shaping the function of CAFs to foster tumor progression.^[^ ^13^ ^]^ For instance, ERS, especially under hypoxia, prompts CAF activation in the tumor microenvironment to induce stromal neovascularization, thereby expediting cancer progression.^[^ ^14^ ^]^ In addition, ERS can induce hypoxic microenvironment and malignant CAF phenotype by mediating the epithelial‐mesenchymal transition process, ultimately accelerating tumor proliferation and invasion.^[^ ^15^ ^]^ Taken together, these data highlight the pivotal regulatory role of ERS‐CAF in chordoma progression, necessitating further mechanistic exploration.

IER2 is a stress‐inducible gene that can be activated in response to various cytokines and noxious stimuli.^[^ ^16^ ^]^ Functioning as a transcriptional regulator, IER2 is involved in diverse cellular processes.^[^ ^17^ ^]^ Previous studies have demonstrated that IER2 expression is increased in activated fibroblasts as well as human colorectal cancer and melanoma tissues, which also correlates with invasive tumor behavior.^[^ ^16^, ^17^ ^]^ Furthermore, recent reports have suggested a close linkage between IER2 and cellular ERS process.^[^ ^18^ ^]^ For instance, it has been proven that IER2 overexpression can suppress HSF1 transcriptional activity via downregulating its phosphorylation level.^[^ ^18^ ^]^ As HSF1 is vital for cellular ER homeostasis and ERS inhibition,^[^ ^19^ ^]^ this observation supports the influence of IER2 on cellular ERS response. Given the crucial role of ERS‐CAF in chordoma progression,^[^ ^8^ ^]^ these findings together imply that IER2 may affect chordoma progression by regulating the ERS process of CAF within the tumor microenvironment. In this study, we determined that microenvironmental hypoxia induced an elevated IER2 expression in CAFs. IER2 overexpression then enhanced the ERS effect of CAF to release GMFG cytokine. The paracrine GMFG signaling from ERS‐CAF promoted tumor cell proliferation, migration, and invasion via binding with ITGB1 on tumor cell, thereby driving chordoma progression. Our study elucidated the precise molecular mechanism of how ERS‐CAF contributed to chordoma progression, which may offer an insight into the development of novel therapeutic strategies possibly by targeting the IER2/GMFG/ITGB1 signaling axis to break the communication between ERS‐CAF and tumor cells.

## 2 Results

### 2.1 Hypoxic Microenvironment Promoted ERS Response in CAFs of Chordoma Tissues

**Figure 1:**
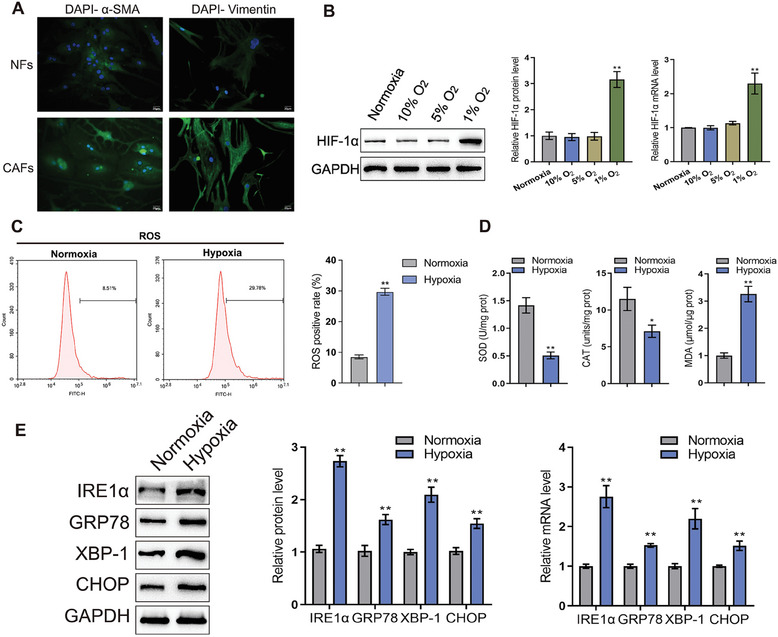
Hypoxic microenvironment promoted ERS response in CAFs of chordoma tissues. A) Representative image of normal fibroblasts (NFs) isolated from normal tissues adjacent to the tumors and cancer‐associated fibroblasts (CAFs) isolated from chordoma tissue; Immunofluorescence staining of α‐SMA and vimentin protein, confirming successful isolation of chordoma CAFs. B) Western blot and qRT‐PCR revealed the expression of hypoxia inducible factor‐1α (HIF‐1α) was significantly upregulated in CAFs under 1% hypoxia condition. C) Flow cytometry revealed the expression levels reactive oxygen species (ROS) was increased in CAF under hypoxic condition. D) Flow cytometry revealed the expression levels of superoxide dismutase (SOD) and catalase (CAT) were reduced, while the expression levels of malondialdehyde (MDA) was increased in CAF under hypoxic condition. E) Western blot and qRT‐PCR revealed ERS related genes (IRE1α, GRP78, XBP‐1, and CHOP) expression was increased in CAF under hypoxic condition.

Hypoxia has been reported to be closely related to tumor progression.^[^ ^20^ ^]^ To investigate the role of hypoxia in chordoma, we first performed GSVA using the bulk RNA‐seq data from 126 tumor samples. The results showed that hypoxic signature score was significantly positively correlated with advanced American Joint Committee on Cancer (AJCC) staging, tumor invasion into surrounding muscles, higher tumor grade, and poor prognosis (Figure S1A–C, Supporting Information). Published reports have suggested that hypoxia could stimulate the cellular ERS process.^[^ ^21^, ^22^ ^]^ Our previous study pinpointed that the hypoxia‐related genes were highly expressed in ERS‐CAF subpopulation.^[^ ^8^ ^]^ Moreover, further GSVA analysis revealed significant positive correlation between ERS‐CAF and hypoxia signature scores in chordoma tissues (Figure S1D, Supporting Information), suggesting that the hypoxic microenvironment might be likely associated with the production of ERS‐CAF. To confirm this hypothesis, we first attempted to isolate the primary CAF from fresh chordoma tissues. After isolation, immunofluorescence identification results showed that α‐SMA and vimentin were highly expressed in the isolated CAFs, while they were lowly expressed in normal fibroblasts (NFs), indicating the successful isolation of primary CAFs (**Figure** **1**A). Subsequently, CAFs were cultured under oxygen concentration gradients of normoxia, 10% O_2_, 5% O_2_, and 1% O_2_, respectively. WB and qPCR results showed that the expression of hypoxia inducible factor‐1α (HIF‐1α) was significantly upregulated in CAFs under 1% hypoxia condition, and the difference was statistically significant compared to the other three subgroups (Figure 1B). Moreover, the expression level of ROS was increased in CAF under hypoxic condition (Figure 1C). Further test revealed that the expression levels of superoxide dismutase (SOD) and catalase (CAT) were reduced, while malondialdehyde (MDA) expression was increased in CAF under hypoxic condition (Figure 1D). In addition, we also found that the expression of several ERS‐related genes (IRE1α, GRP78, XBP‐1, and CHOP) was elevated in CAF under hypoxic condition (Figure 1E). These findings collectively indicated that the hypoxic microenvironment could activate the ERS response of CAF and thus induced its phenotypic transition to ERS‐CAF.

### 2.2 ERS‐CAF Promoted the Malignant Progression of Chordoma

**Figure 2:**
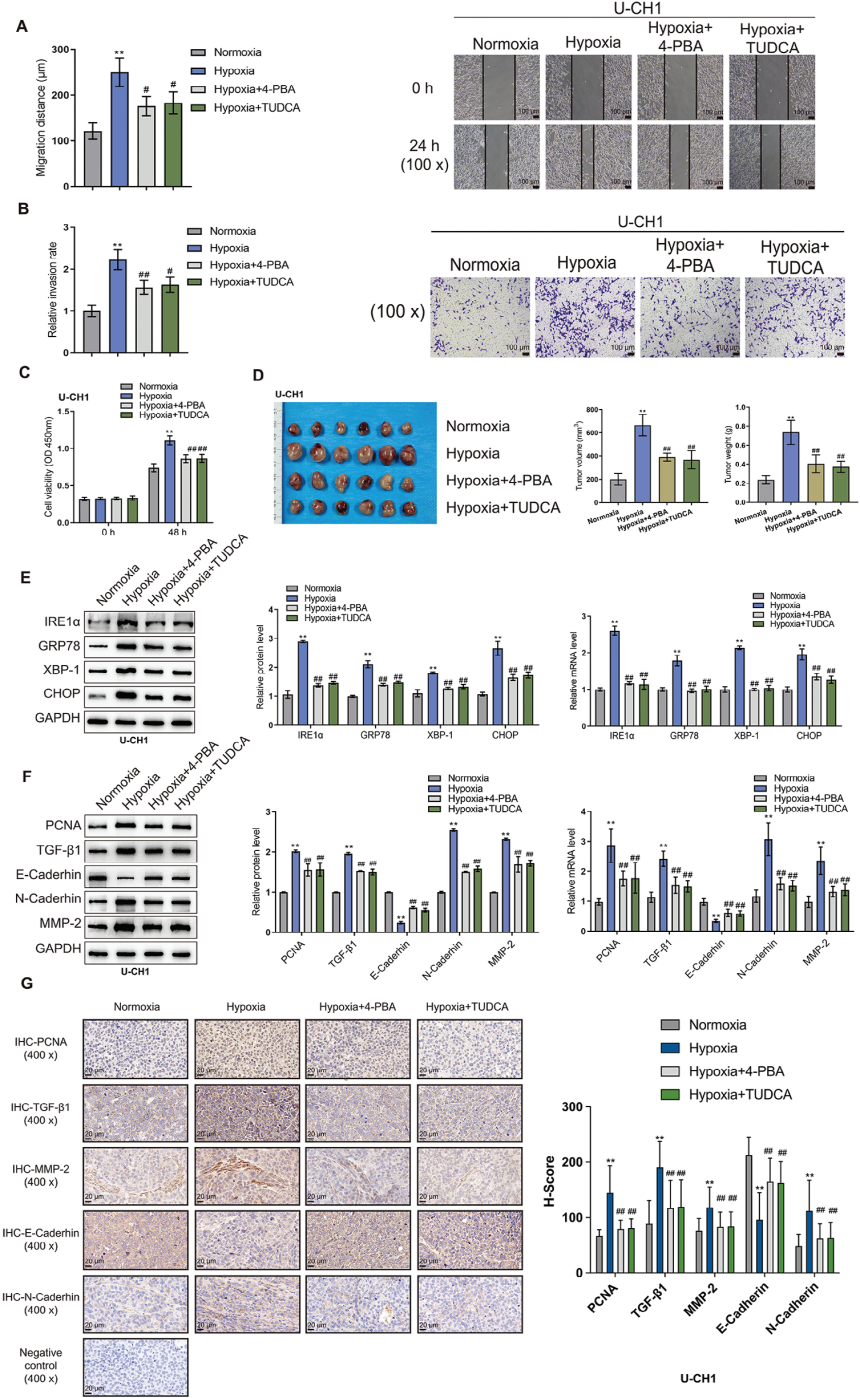
ERS‐CAF promoted the malignant progression of chordoma. A) Wound healing assay shows enhanced migration ability of U‐CH1 chordoma cells co‐cultured with endoplasmic reticulum stress‐related CAF (ERS‐CAF), while blocking ERS response of CAF with 4‐phenylbutyric acid (4‐PBA) and tauroursodeoxycholic acid (TUDCA) reversed the above phenomenon. B) Transwell assay reveals increased invasion ability of U‐CH1 chordoma cells co‐cultured with ERS‐CAF, while blocking ERS response of CAF with 4‐PBA and TUDCA reversed the above phenomenon. C) Cell Counting Kit‐8 (CCK8) assay demonstrates enhanced proliferation activity of U‐CH1 chordoma cells co‐cultured with ERS‐CAF, while blocking ERS response of CAF with 4‐PBA and TUDCA reversed the above phenomenon. D) Macroscopic image of subcutaneous tumor formation in mice, indicating increased tumor weight and volume after co‐transplantation with U‐CH1 chordoma cells and ERS‐CAF, while blocking ERS response of CAF with 4‐PBA and TUDCA reversed the above phenomenon. E) Western blot (WB) and qRT‐PCR revealed ERS related genes expression was increased in subcutaneous tumor samples after co‐transplantation with U‐CH1 chordoma cells and ERS‐CAF, while blocking ERS response of CAF with 4‐PBA and TUDCA reversed the above phenomenon. F) WB and qRT‐PCR revealed EMT related proteins (including PCNA, TGF‐B1, N‐cadherin, and MMP‐2) expression were increased in subcutaneous tumor samples after co‐transplantation with U‐CH1 chordoma cells and ERS‐CAF, while blocking ERS response of CAF with 4‐PBA and TUDCA reversed the above phenomenon. G) Immunohistochemical analysis of tumor samples showed high expression of EMT‐related genes after co‐transplantation with U‐CH1 chordoma cells and ERS‐CAF, while blocking ERS response of CAF with 4‐PBA and TUDCA reversed the above phenomenon.

To further investigate the impact of ERS‐CAF on the biological behavior of chordoma, we co‐cultured CAF with the U‐CH1 cell line. Cell Counting Kit‐8 (CCK8), wound healing, and transwell assays revealed that CAF treated by hypoxia intervention significantly enhanced the proliferation, migration, and invasion ability of chordoma cells (**Figure** **2**A–C), while blocking ERS response of CAF with 4‐phenylbutyric acid (4‐PBA) and tauroursodeoxycholic acid (TUDCA) reversed the above phenomenon (Figure 2A–C). The similar results were observed when CAFs were co‐cultured with the UM‐Chor1 cell line (Figure S2A–C, Supporting Information). Subsequently, we co‐transplanted CAF and U‐CH1 cells in a 1:1 ratio before and after hypoxic treatment into subcutaneous tissue of mice. Our analyses showed that the volume and weight of the tumors in the hypoxic treatment group were larger than those in the normal group (Figure 2D), which was also the case for expression levels of ERS related genes in these tumors (Figure 2E). In contrast, intervention with 4‐PBA and TUDCA inhibited the tumor progression (Figure 2D,E). We also observed the similar outcomes when CAFs and UM‐Chor1 cells were co‐implanted into the subcutaneous tissue of mice at a 1:1 ratio before and after hypoxic treatment (Figure S2D,E, Supporting Information).

It has been reported that EMT is closely related to tumor invasion and metastasis, and CAF can accelerate disease progression by inducing EMT in tumor cells.^[^ ^23^ ^]^ In the in vitro co‐culture models with U‐CH1 or UM‐Chor1 cells, we found that hypoxia‐treated CAF could induce the expression of EMT related proteins (including PCNA, TGF‐B1, N‐cadherin, and MMP‐2) on tumor cells, while reducing the expression abundance of E‐cadherin (Figure 2F; Figure S2F, Supporting Information). Similar results were also observed in immunohistochemical analysis of subcutaneous tumor samples in vivo (Figure 2G; Figure S2G, Supporting Information). These data together indicated that ERS‐CAF induced by hypoxia condition could aggravate the malignant progression of chordoma.

### 2.3 Hypoxic Microenvironment Promoted Chordoma Progression by Inducing IER2‐Mediated Phenotypic Shift of CAF toward ERS Subtype

**Figure 3:**
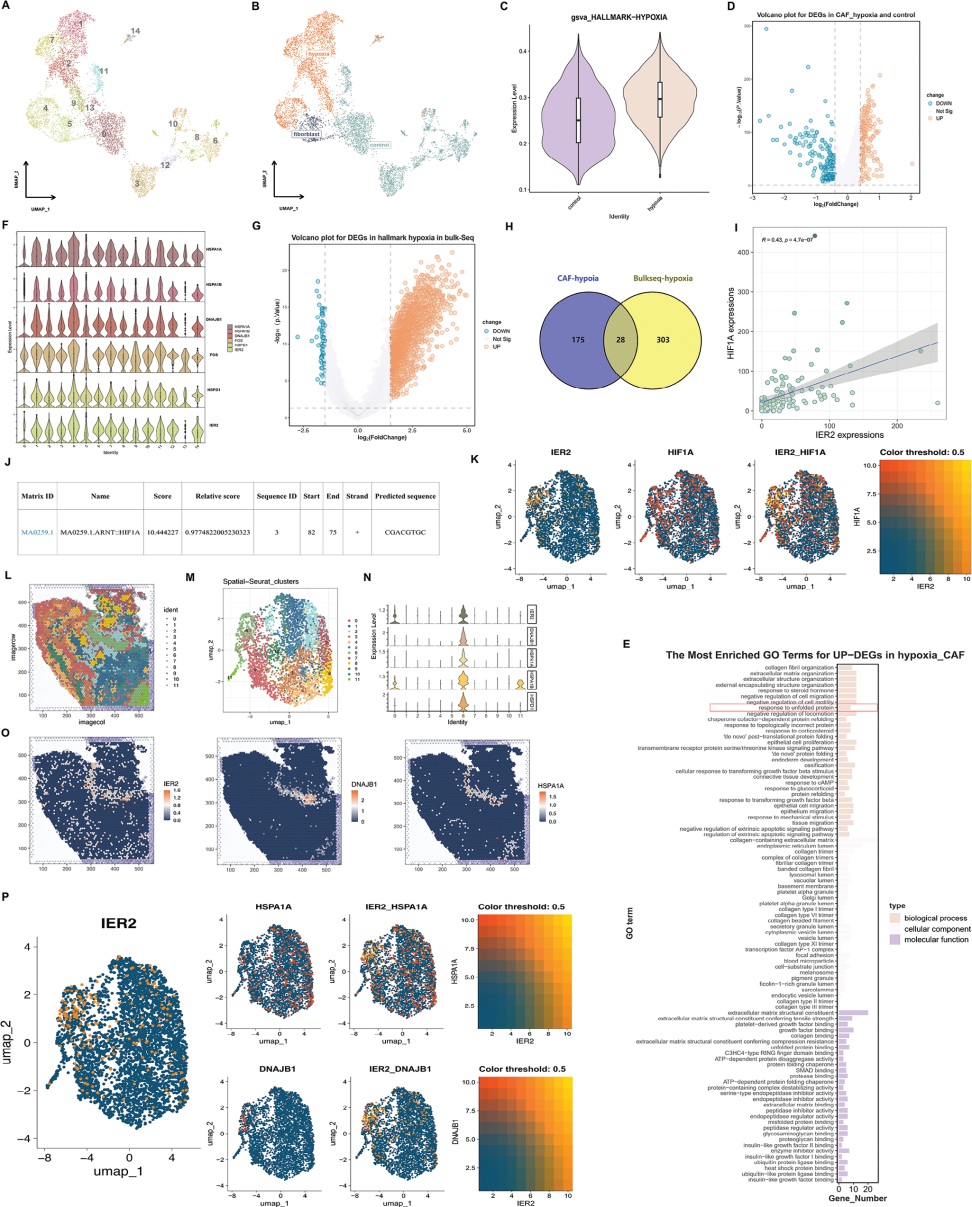
IER2 was associated with the hypoxic microenvironment in chordoma. A) UMAP projection of 15 fibroblast clusters across all samples. B) UMAP plot of normal fibroblasts, control CAFs, and hypoxic CAFs. C) Hypoxic CAFs had higher GSVA‐HALLMARK‐Hypoxia scores than control CAFs. D)Volcano plots for DEGs in hypoxic CAFs and control CAFs. E) GO enrichment analysis of up‐DEGs in hypoxic CAFs. F) Violin plot showing the expression levels of ERS‐CAF related DEGs for each fibroblast subpopulation. G) Volcano plots for DEGs in HALLMARK‐Hypoxia in bulk RNA‐seq data. H) DEGs upregulated in hypoxic CAFs from the scRNA‐seq data were intersected with DEGs from tumors with high hypoxic signature scores from the Bulk RNA‐seq data, identifying 28 overexpressed DEGs in CAFs induced by hypoxic conditions. I) Correlation between IER2 and HIF1A expression of Bulk RNA‐seq data. J) Using the eukaryotic promoter database prediction, a HIF1A high‐binding site was obtained at 85 bp upstream of the IER2 promoter. K) Spatial transcriptome (ST) data demonstrate strong spatial co‐expression between IER2 and HIF1A with significant spatial co‐expression coefficient. Color ranges blue (low) to yellow (high) represents correlation score. L) ST representation of chordoma tissue. M) After Seurat dimensionality reduction clustering analysis, 12 spatial clusters were obtained. N) Violin plot showing the expression levels of ERS‐CAF‐related DEGs in spatial cluster 5, indicating that spatial cluster 5 represents ERS‐CAF. O) Correlation between the spatial positions of IER2 and ERS‐CAF marker genes such as HSPA1A and DNAJB1. P) ST data showed strong spatial co‐expression between IER2 and ERS‐CAF marker genes such as HSPA1A and DNAJB1, with significant spatial co‐expression coefficients. Color ranges blue (low) to yellow (high) represent correlation score.

**Figure 4:**
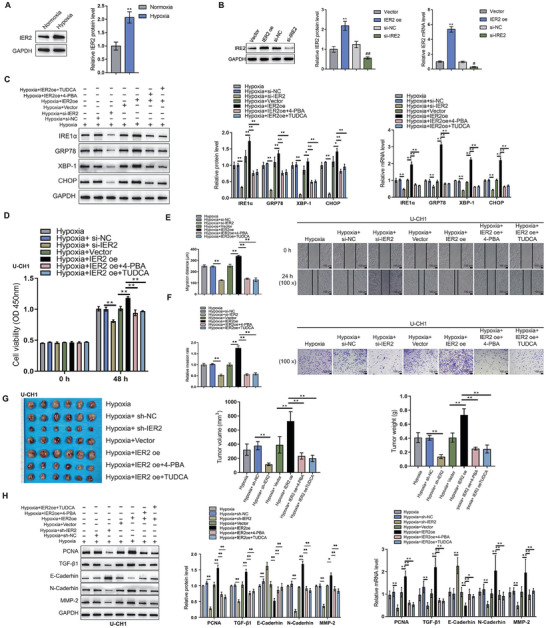
IER2 mediated CAF phenotypic transition to ERS subtype and promotes chordoma progression. A) WB and qPCR detection showed that IER2 was significantly overexpressed in chordoma CAF after hypoxia treatment. B) Establishment of IER2 knockdown and overexpression CAF cell lines. C) WB and qRT‐PCR showed that IER2 overexpression enhanced the ERS effect of CAF under hypoxic conditions, and IER2 knockdown or anti‐ERS blocker intervention could reverse this phenomenon. D) CCK8 assay demonstrates enhanced proliferation activity of U‐CH1 chordoma cells co‐cultured with CAFs having IER2 overexpression, while genetic knockdown of IER2 in CAF and administering with anti‐ERS agents reversed the above phenomenon. E) Wound healing assay shows enhanced migration ability of U‐CH1 chordoma cells co‐cultured with CAFs having IER2 overexpression, while genetic knockdown of IER2 in CAF and administering with anti‐ERS agents reversed the above phenomenon. F) Transwell assay reveals increased invasion ability of U‐CH1 chordoma cells co‐cultured with CAFs having IER2 overexpression, while genetic knockdown of IER2 in CAF and administering with anti‐ERS agents reversed the above phenomenon. G) Macroscopic image of subcutaneous tumor formation in mice, indicating increased tumor weight and volume after co‐transplantation with U‐CH1 chordoma cells and CAFs having IER2 overexpression, while genetic knockdown of IER2 in CAF and administering with anti‐ERS agents reversed the above phenomenon. H) WB and qRT‐PCR revealed EMT related proteins expression were increased in subcutaneous tumor samples after co‐transplantation with U‐CH1 chordoma cells and CAFs having IER2 overexpression, while genetic knockdown of IER2 in CAF and administering with anti‐ERS agents reversed the above phenomenon.

To gain insights into the specific mechanism underlying ERS‐CAF formation, we first conducted analyses on single‐cell RNA‐sequencing (scRNA‐seq) data. We retrieved 92004 individual cells in total, including 81602 cells from 9 chordoma samples and 10 402 cells from 5 nucleus pulposus (NP) samples (as controls). A total of 25 cellular subgroups were identified (Figure S3A, Supporting Information). Through markers identification, fibroblasts, mural cells, endothelial cells, epithelial‐like cells (malignant cells), myeloid cells, T cells, B cells, granulocytes, and chondrocytes were determined (Figure S3B, Supporting Information). Among them, cluster 4 and cluster 19 were identified as fibroblasts (Figure S3A,B, Supporting Information). Following this, we then performed a dimensionality reduction of these 7220 fibroblasts, resulting in the identification of 15 subclusters (**Figure** **3**A). Through calculation of the hypoxic signature score obtained by GSVA, we found that subcluster 5 corresponded to normal fibroblasts, while the others represented CAFs (Figure 3B,C), with subcluster 1, 2, 4, 7, 9, and 14 being defined as hypoxic CAFs (Figure 3B). Furthermore, the differentially expressed genes (DEGs) for hypoxic CAF were functionally enriched in ERS‐reated signaling pathway, such as “response to unfolded protein” (Figure 3D,E). In this analysis, as subcluster 4 displayed significantly high expression of ERS‐CAF related DEGs (Figure 3F),^[^ ^8^ ^]^ we thus defined this cell cluster as ERS‐CAF and found that it was totally included in the hypoxic CAF subpopulation (Figure 3A,B). These results indicated that hypoxic microenvironment may promote the formation of the ERS‐CAF. To further probe the regulatory mechanism, we then intersected the upregulated DEGs in hypoxic CAF from scRNA‐seq data and those in tumors with high hypoxic signature scores in bulk RNA‐seq data, and identified 28 overexpressed DEGs in CAFs induced by hypoxic condition (Figure 3G,H). Among them, we focused on stress‐related transcription factors (TFs) considering they could possibly represent the upstream regulatory molecules promoting the formation of ERS‐CAF in hypoxic environment. As HIF‐1α is well known to play a crucial role in response to hypoxia, we, therefore, detected the relationship between the expression of these TFs and HIF‐1α in chordoma tissues using the bulk RNA‐seq data. We found significant correlation between IER2 and HIF‐1α expression (Figure 3I). Moreover, we obtained a high binding site of HIF‐1α at 85 bp upstream of the IER2 promoter by prediction using the eukaryotic promoter database (Figure 3J). Further spatial transcriptomic (ST) data from six tumor samples indicated a significant spatial co‐expression between IER2 and HIF‐1α (Figure 3K). Moreover, ST data showed that the ERS‐CAF (cluster 6) harbored specifically high expression of IER2 (Figure 3L–O), and there was also a remarkable spatial co‐localization between specific marker genes of ERS‐CAF and IER2 as assessed by spatial co‐expression coefficient (Figure 3O,P). In addition, by analyzing the bulk RNA‐seq data, we unveiled that high expression of IER2 was positively correlated with high pathological grade, tumor invasion of surrounding muscle tissues, advanced AJCC staging as well as poor overall survival and local recurrence‐free survival of patients (Figure S3C,D, Supporting Information). As bulk RNA‐seq cannot delineate gene expression at the cellular level, we further attempted to accurately define the clinical relevance of stromal IER2 expression in 105 patients using multiplexed quantitative immunofluorescence (QIF) staining (Figure S4A, Supporting Information). We found that IER2^+^ ERS‐CAF density was significantly correlated with the invasive tumor phenotype (such as AJCC staging, high pathological grade, and invasion of surrounding muscle) and poor patient clinical outcomes (Figure S4B,C, Supporting Information). Previous studies have demonstrated that IER2 is upregulated in activated fibroblasts and can trigger the cellular ERS process.^[^ ^16^, ^18^ ^]^ These data suggest that IER2 may likely contribute to the presence of ERS‐CAF subtype. To confirm this speculation, subsequent WB and qPCR detection showed that IER2 was significantly overexpressed in CAF after hypoxic treatment (**Figure** **4**A). To further evaluate the regulatory effect of IER2 on ERS status in CAFs, we successfully constructed CAF cells with IER2 knockdown and overexpression (Figure 4B). WB and qPCR experiments revealed that IER2 overexpression enhanced the ERS effect of CAF under hypoxic condition, while IER2 knockdown or intervention with anti‐ERS blockers could reverse this phenomenon (Figure 4C). The above results hinted at that hypoxia reprogramed CAF into ERS‐CAF subtype via upregulation of IER2.

To dissect the functional role of IER2 on CAF, we co‐cultured CAFs having IER2 knockdown or overexpression with U‐CH1 or UM‐Chor1 cell lines. Our analyses showed that CAF with IER2 overexpression significantly augmented the proliferation, migration, and invasion ability of U‐CH1 and UM‐Chor1 cells (Figure 4D–F; Figure S5A–C, Supporting Information), while genetic knockdown of IER2 in CAF and administering with anti‐ERS agents impeded the effects of IER2 overexpression. In addition, in the in vivo subcutaneous tumorigenesis model, the weight and volume of tumors were significantly higher in mice bearing CAF with IER2 overexpression than those in the controls (Figure 4G; Figure S5D, Supporting Information). Similar outcomes were also seen for the expression levels of EMT related factors in these tumor tissues (Figure 4H; Figure S5E, Supporting Information). However, use of anti‐ERS drugs could effectively reverse these findings (Figure 4D–H; Figure S5A–E, Supporting Information).

### 2.4 Hypoxic Upregulation of IER2 Increased Paracrine GMFG Signaling from ERS‐CAF to Promote Chordoma Progression

As a transcription regulatory factor, IER2 plays an important role in various cellular responses and can impact the invasive behavior of tumors.^[^ ^16^, ^17^ ^]^ Studies have pointed out that CAF regulates the biological behavior of tumor cells mainly through paracrine signaling.^[^ ^24^ ^]^ To probe the downstream mechanism behind ERS‐CAF's promotion of chordoma progression, we collected supernatants of CAF before and after IER2 knockdown for data‐independent acquisition proteomics analysis. Differential expression analysis revealed that GMFG was the most significantly altered secretory protein (Figure S6A, Supporting Information). Besides, ELISA assay validated that overexpression of IER2 dramatically increased the abundance of GMFG in CAF supernatant (Figure S6B, Supporting Information). Conversely, IER2 knockdown significantly diminished the level of GMFG in CAF supernatant (Figure S6B, Supporting Information). Collectively, these findings implied that IER2 upregulation prompted paracrine GMFG secretion by ERS‐CAF.

To further determine the biological function of GMFG, we silenced GMFG in CAF while introducing exogenous GMFG to CAF culture media to observe their impact on tumor biology. Our results showed that exogenous GMFG factor significantly enhanced the proliferation, migration, and invasion ability of U‐CH1 and UM‐Chor1 cells, while GMFG silence on CAF mitigated tumor cell malignancy (Figures S6C–E and S7A–C, Supporting Information). In vivo subcutaneous tumorigenesis experiments also unveiled that local injection of exogenous GMFG significantly promoted tumor growth and proliferation (Figures S6F and S7D, Supporting Information). Tumor tissues from this group also exhibited markedly elevated levels of EMT‐associated marker genes (Figures S6G and S7E, Supporting Information). Furthermore, tissue staining using Trichrome and Picrosirius Red demonstrated a high content of type I collagen and collagen fibers in tumors with exogenous GMFG intervention (Figures S6H and S7F, Supporting Information).

### 2.5 Paracrine GMFG Signaling from ERS‐CAF Accelerated Chordoma Progression by Positively Targeting ITGB1

**Figure 5:**
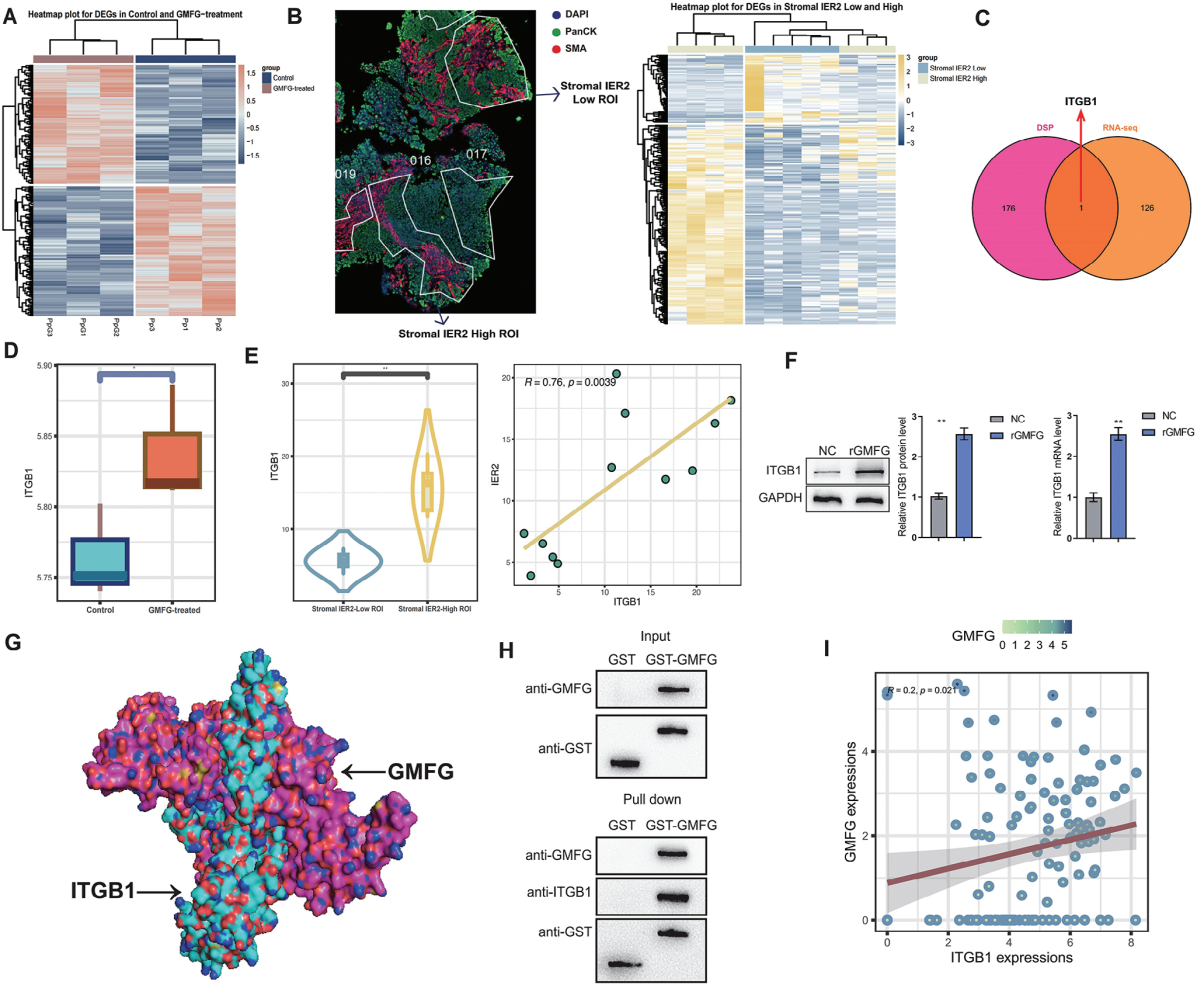
ERS‐CAF mediates the paracrine secretion of GMFG targeting ITGB1. A) Heatmap of DEGs for mRNA expression profiles of chordoma cells before (control) and after incubation with exogenous GMFG (GMFG‐treated). B) GeoMx Digital Spatial Profiler (DSP) transcriptome sequencing of 10 regions of interest (ROIs) with high (n = 5) and low (n = 5) expression of stromal IER2. C) ITGB1 is the only target gene common to the up‐regulated genes in both cell line mRNA sequencing and DSP sequencing. D) ITGB1 was significantly up‐regulated in the GMFG‐treated group. E) ITGB1 was significantly increased in ROIs with high expression of stromal IER2, and the expression of IER2 in ROIs was significantly positively correlated with the expression of ITGB1. F) WB and qRT‐PCR demonstrated that exogenous GMFG treatment significantly induced ITGB1 expression in tumor cells. G) Molecular docking demonstrated the existence of binding sites for ITGB1 and GMFG. H) GST pull‐down assay confirmed the existence of a targeted binding site between GMFG and ITGB1. I) Bulk RNA‐seq data demonstrate a significant positive correlation between GMFG and ITGB1 expression.

To unveil GMFG's downstream targets in regulating chordoma progression, we used RNA‐seq to investigate the mRNA expression profiles of chordoma cells before and after incubation with exogenous GMFG. Based on differential analysis and screening criteria, a total of 309 DEGs were identified, of which 127 were upregulated and 182 were downregulated (**Figure** **5**A). In addition, we also conducted transcriptomic sequencing on ten regions of interest (ROIs, each containing both tumor and stromal cells) with high (n = 5) or low (n = 5) stromal IER2 expression using GeoMx Digital Spatial Profiler (DSP) method. Differential expression analysis resulted in 237 DEGs, including 177 upregulated and 60 downregulated genes (Figure 5B). By intersecting the upregulated genes from mRNA sequencing of cell lines with those from DSP sequencing, we identified ITGB1 as the only target gene among the upregulated genes in both groups (Figure 5C). Further post‐hoc analysis validated that ITGB1 was truly significantly up‐regulated in tumor cells after the exogenous GMFG intervention (Figure 5D). Similarly, elevated tumoral ITGB1 expression was also observed in ROI with high stromal IER2 expression compared to their counterparts, and the expression of IER2 and ITGB1 was significantly positively correlated among all ROIs selected for analyses (Figure 5E). Subsequent qPCR and WB analysis evidenced that exogenous GMFG treatment markedly induced ITGB1 expression on tumor cells (Figure 5F). Molecular docking analysis also demonstrated the existence of binding sites for ITGB1 and GMFG (Figure 5G). Further, GST pull‐down assay confirmed the targeted binding sites between GMFG and ITGB1 (Figure 5H). Moreover, we identified a significant positive correlation between GMFG and ITGB1 expression based on the bulk RNA‐seq data from 126 chordoma samples (Figure 5I). Additionally, we conducted correlation analysis between ITGB1 and GMFG using two chordoma bulk RNA‐seq datasets (GSE239531 and GSE205457) from the GEO database as well as RNA‐seq data involving 42 bone tumor cell lines from the CCLE database. The results consistently showed a significant relationship among these two genes (Figure S8A, Supporting Information). These data indicated that GMFG exerted its biological functions by targeting ITGB1 on tumor cells.

To examine the clinical significance ITGB1 in chordoma, we assessed the relationship between their expression and patient outcomes using bulk RNA‐seq data involving 126 tumor samples. Patients were divided into high expression and low expression subgroups according to the Cutoff Finder Web Application. Our analyses unveiled that high expression of ITGB1 was positively correlated with tumor invasion into surrounding muscle tissues and advanced AJCC staging (Figure S8B, Supporting Information). Kaplan–Meier curves showed that ITGB1 expression was significantly related to poor overall survival and local recurrence‐free survival of patients (Figure S8C, Supporting Information). Further, QIF experiments confirmed that ITGB1 expression on tumor cells was significantly correlated with tumor aggressiveness and patient clinical outcomes (Figure S4D, Supporting Information). Noticeably, our analysis also disclosed that IER2^+^ ERS‐CAF displayed the most proximity to ITGB1^+^ tumor cells, mainly enriched within a 60 µm radius of ITGB1^+^ tumor cells (Figure S9, Supporting Information).

To clarify the regulatory role of the GMFG/ITGB1 signaling axis in chordoma progression, we introduced exogenous GMFG to the CAF medium. These CAFs were co‐cultured with U‐CH1 or UM‐Chor1 cells featuring ITGB1 knockdown (Figures S10A and S11A, Supporting Information) to observe the alterations in tumor cell behavior and ITGB1 signaling. Our analyses unveiled that exogenous GMFG promoted ITGB1 expression on chordoma tumor cells (Figures S10B and S11B, Supporting Information). Notably, ITGB1 knockdown attenuated the proliferation, migration, and invasion of U‐CH1 and UM‐Chor1 cells, which could be partially reversed through exogenous GMFG addition (Figures S10C–E and S11C–E, Supporting Information). Furthermore, in vivo tumorigenic experiments uncovered that ITGB1 knockdown substantially repressed tumor growth and proliferation. This effect was accompanied by the decrease in expression level of EMT‐related genes, as well as type I collagen and collagen fiber content. Remarkably, local injection of exogenous GMFG could reverse this phenomenon (Figures S10F–H and S11F–H, Supporting Information). Taking together, these outcomes suggested that ERS‐CAF‐derived GMFG drove chordoma malignant progression through its interaction with ITGB1 on tumor cells.

### 2.6 GMFG/ITGB1 Axis Might Regulate the Biological Behavior of Chordoma by Mediating Immunosuppression in the Tumor Microenvironment

**Figure 6:**
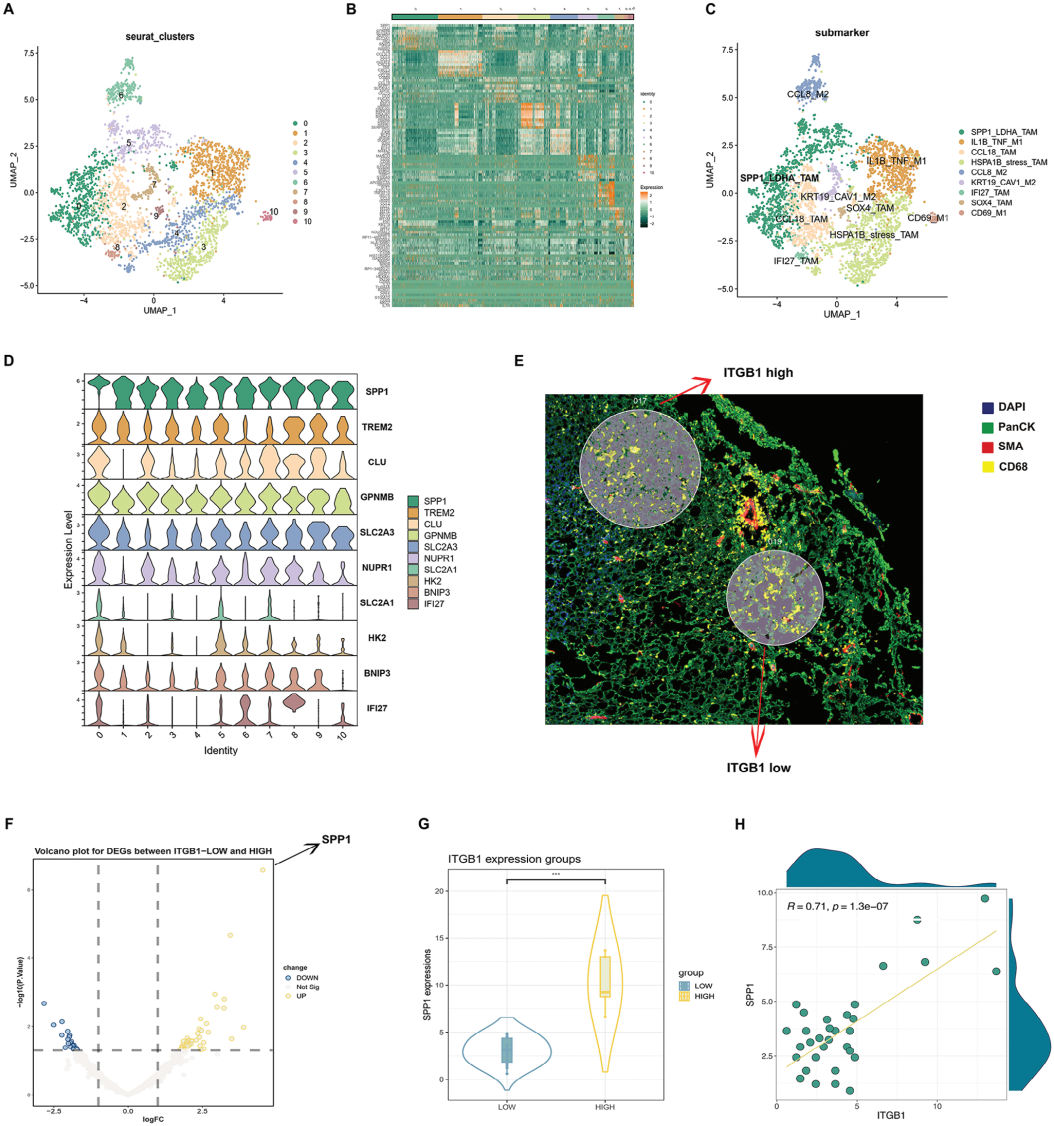
GMFG/ITGB1 axis might regulate the biological behavior of chordoma by mediating immunosuppression in the tumor microenvironment. A) UMAP projection of 11 macrophages clusters across all samples. B) Heatmap of top 10 DEGs per cluster for 11 macrophages clusters. C) UMAP projection of macrophage clusters named after specific marker genes in all samples. D) Violin plot showing the expression levels of 11 macrophages clusters, SPP1 displayed specifically high expression in tumor‐associated macrophage subcluster 0 and subcluster 5. E) GeoMx DSP transcriptome sequencing of 42 ROIs with high (n = 37) and low (n = 5) expression of tumoral ITGB1 expression. F) Volcano plots for DEGs in tumoral ITGB1 high expression ROIs and low expression ROIs. G) SPP1 was significantly upregulated in ROIs with high tumor ITGB1 expression. H) DSP data analysis showed that the expression of SPP1 and ITGB was significantly positively correlated.

To delve into the biological behavior regulated by the GMFG/ITGB1 axis that affected the progression of chordoma, we first reanalyzed the bulk RNA‐seq data (n = 126). We uncovered that hypoxic and ERS‐CAF signature scores as well as the expression of IER2 were significantly positively correlated with the immune cells abundance (especially macrophage) within the chordoma microenvironment (Figure S12A–E, Supporting Information). These data hint at that the downstream GMFG/ITGB1 signaling may possibly function by modulating the functions of macrophages infiltrated in chordoma microenvironment. To validate this hypothesis, we conducted further interaction analysis using scRNA‐seq data. After dimensionality reduction of myeloid cells, we obtained 23 subclusters (Figure S13A, Supporting Information), among which macrophage markers were specifically expressed in subcluster 5 (Figure S13B,C, Supporting Information). Notably, we disclosed a significantly strong interaction between tumor cells with high ITGB1 expression (ITGB1^high^) and the macrophages (Figure S13D, Supporting Information). To further clarify the interaction between macrophage subsets and ITGB1^high^ tumor cells, we then performed re‐dimensionality reduction analysis on macrophages and inferred 11 tumor‐associated macrophage (TAM) subpopulations (**F** **igure 6**A,B). Among them, SPP1 displayed specifically high expression in TAM subcluster 0 and subcluster 5 (SPPI^+^ TAM) (Figure 6C,D). Further cell‐cell communication analysis using CellChat revealed the strongest interaction between SPP1^+^ TAM and ITGB1^high^ tumor cells (Figure S14, Supporting Information). Additionally, we utilized the DSP method to perform transcriptome sequencing on 42 ROIs (each containing both tumor cells and macrophages) with high (n = 5) and low (n = 37) tumoral ITGB1 expression (Figure 6E). Differential expression analysis revealed 20 significantly downregulated and 41 significantly upregulated DEGs. Among them, SPP1 was significantly upregulated on macrophages in ROIs with high tumor ITGB1 expression, and the expression of these two genes was significantly positively correlated by analyzing the DSP data (Figure 6F–H). It has been reported that SPP1^+^ TAMs exhibit immunosuppressive characteristics, indicating poor prognosis in various types of cancer.^[^ ^25^, ^26^ ^]^ Furthermore, previous observations have confirmed that chordomas have an inhibitory immune microenvironment, where the density of immune cells significantly affects disease progression.^[^ ^8^, ^27^, ^28^ ^]^ Taking together, we speculate that GMFG may potentially mediate biological functions through activating the SPP1/ITGB1 pathway to induce immunosuppression in the microenvironment.

To further confirm the interactive SPP1/ITGB1 signaling between TAMs and tumor cells, we conducted QIF analysis on another independent cohort involving 105 tumor tissue samples (Figure S15A, Supporting Information). We found that the expression of SPP1/ITGB1 signaling was more pronounced in the regions of tumor cells proximal to TAMs (Figure S15B,C, Supporting Information). Collectively, these data suggest that ITGB1 on tumor cells might likely expedite chordoma progression by inducing an inhibitory immune microenvironment through recruiting SPP1^+^ TAMs, although the precise molecular mechanisms deserve further exploration.

## 3 Discussion

The present study established the IER2 overexpression induced by hypoxic microenvironment as a pivotal prerequisite for CAF's transformation to the ERS phenotype. IER2 stimulated GMFG factor secretion by ERS‐CAF, which then promoted chordoma malignant progression through binding with ITGB1 on tumor cells. Importantly, interventions targeting GMFG signaling could disrupt the communication between ERS‐CAFs and tumor cells, and thus inhibit ERS‐CAF's cancer‐promoting function. These data unveil the precise molecular mechanisms underlying ERS‐CAF's role in chordoma progression, which may offer novel insights and targets for the development of future therapeutic strategies.

Our earlier study revealed that ERS‐CAF could contribute to the malignant advancement of chordoma,^[^ ^8^ ^]^ yet the specific mechanism remains elusive. Considering the pronounced expression of hypoxic genes in ERS‐CAF and the important influence of hypoxia on tumorigenesis as well as cellular ERS effect within the tumor microenvironment,^[^ ^15^, ^29^, ^30^, ^31^ ^]^ we, therefore, speculated that hypoxia played a key role in the cancer‐promoting process of ERS‐CAF. In line with this, our investigation illustrated that hypoxia‐triggered ERS activation in CAF could intensify its pro‐carcinogenic impact. In addition, our analyses indicated a crucial role for hypoxia‐induced IER2 upregulation in ERS‐CAF formation, which was also closely linked to clinical features and prognosis of chordoma. IER2, a stress gene, serves as a transcriptional regulator in various cellular responses.^[^ ^16^ ^]^ Published data have shown hypoxia‐induced IER2 upregulation in activated fibroblasts^[^ ^16^, ^32^, ^33^ ^]^ and significant correlation of IER2 mRNA levels with cancer progression.^[^ ^16^, ^17^ ^]^ Recent reports highlight IER2's role in inducing cellular ERS through the MAPK/ERK signaling,^[^ ^34^, ^35^ ^]^ thereby enhancing the proliferative and invasive ability of colorectal cancer and melanoma cells.^[^ ^16^, ^17^ ^]^ Altogether, these data reinforce our observation that hypoxia‐induced IER2 upregulation to activate the ERS response in CAFs represents a pivotal biological process in chordoma progression. Given CAFs' genetic uniformity and lower drug resistance compared to tumor cells,^[^ ^36^ ^]^ these findings may lay the foundation for developing future therapeutic strategies by targeting ERS‐CAF through interventions with IER2 blockade or ERS inhibitors.

Prior reports have demonstrated CAF's modulation of tumor behavior via a paracrine manner.^[^ ^24^, ^37^ ^]^ Similarly, our study corroborated that ERS‐CAF affected chordoma cell malignancy through paracrine GMFG cytokine. Moreover, GMFG inhibition markedly suppressed tumor progression. As a vital ADF/cofilin protein superfamily member, GMFG is key regulator of actin cytoskeleton reorganization.^[^ ^38^ ^]^ Researchers have found that GMFG is of significance in angiogenesis and knockdown of this gene can severely hinder intersegmental vessel formation in zebrafish.^[^ ^39^ ^]^ In the cancer realm, it has been reported that GMFG functions as a critical oncogene in mediating proliferation and invasion of ovarian and breast cancer cells. Furthermore, GMFG expression is also shown to be associated with infiltration by diverse suppressive immune cells.^[^ ^40^, ^41^ ^]^ Supporting these, our in vivo and in vivo outcomes uncovered GMFG's role in promoting chordoma progression. These results may pave the way for exploring new therapeutic targets (such as GMFG) in chordoma, though further exploration is essential.

To further understand the functional role of GMFG in chordoma progression, we then employed bulk RNA‐seq and DSP techniques to identify GMFG‐mediated downstream signaling pathways. Our analyses indicated that GMFG exerted its biological function through interaction with ITGB1 on tumor cells. Integrin β1 (ITGB1), a transmembrane receptor that mediates the interaction between cells and the extracellular environment, is a member of the integrin protein family. ITGB1 overexpression has been observed in many human malignancies, including lung, breast, and colorectal cancers, significantly impacting metastasis and patient survival.^[^ ^42^, ^43^ ^]^ Consistent with these data, we observed a significant correlation of ITGB1 expression with chordoma characteristics and patient outcomes. Besides, overexpression of ITGB1 led to increased tumor cell invasion. The exact mechanisms of how the GMFG/ITGB1 signaling influences chordoma biology is not known. Previous reports have suggested that GMFG can enhance invasion and metastasis of tumor cells by regulating the stability of ITGB1 molecule to induce migration of myeloid‐derived suppressor cells into the tumor microenvironment.^[^ ^44^ ^]^ Additionally, both GMFG and ITGB1 expressions are reported to correlate with the densities of microenvironmental immune cells.^[^ ^45^, ^46^ ^]^ Our subsequent analyses hinted at a spatial relationship between ITGB1^+^ tumor cells and SPP1^+^ TAMs, which were both related to clinical data of patients. Preceding observations have verified a suppressive immune microenvironment and its significant influence on chordoma behavior and outcomes.^[^ ^6^, ^47^ ^]^ Altogether, these data suggest that the GMFG/ITGB1 axis may likely accelerate chordoma malignancy by inducing migration of SPP1^+^ TAMs to reshape an inhibitory immune milieu. Therefore, targeting ERS‐CAF may represent a new immunotherapy strategy, which is also supported by a recent study showing that ERS‐CAF is associated with microenvironmental immune cells infiltration and response to immunotherapy in patients with skull base chordoma.^[^ ^48^ ^]^ Future molecular explorations are warranted to further validate this conjecture.

## 4 Conclusion

The current study demonstrated that the hypoxic chordoma microenvironment could induce sustained expression of IER2 in CAF to stimulate its ERS effect and then released GMFG cytokine. GMFG produced by ERS‐CAF mediated the formation of an inhibitory immune microenvironment to promote tumor cell proliferation, migration, and invasion by binding to ITGB1 on tumor cells, ultimately leading to chordoma progression. Our study elucidated the specific molecular mechanism by which ERS‐CAF regulated the progression of chordoma, which may provide novel insights and potential targets for the development of ERS‐CAF‐based therapeutic approaches for this rare disease.

## 5 Experimental Section

### Patients and Tissue Samples

This study was approved by the local ethical committee of the hospital and informed written consents were obtained from all patients. Please see Supplementary Methods and Table S1 (Supporting Information) for details.

### Cell Line

The U‐CH1 and UM‐Chor1chordoma cell lines were acquired from American Type Culture Collection (ATCC). Please see Supplementary Methods for details.

### Isolation and Culture of Primary Fibroblasts

CAFs and normal fibroblasts (NFs) were isolated from chordoma and adjacent normal tissues, respectively. Please see Supplementary Methods for details.

### Preparation of Conditioned Medium (CM)

Please see Supplementary Methods for details.

### Indirect Co‐Culture

CAFs were seeded in cell culture inserts. Please see Supplementary Methods for details.

### Cell Proliferation Assay

Cell growth was assessed using the Cell Counting Kit‐8 (CCK8) assay. Please see Supplementary Methods for details.

### Wound‐Healing Assay

The migratory ability of chordoma cells was evaluated using the Wound‐healing assay. Please see Supplementary Methods for details.

### Transwell Migration and Invasion Assays

The invasion ability of chordoma cells was evaluated in transwell chamber. Please see Supplementary Methods for details.

### Detection of Reactive Oxygen Species (ROS) Levels in CAFs via Flow Cytometry

The cells were subjected to staining using the ROS kit. Please see Supplementary Methods for details.

### Western Blotting

Please see Supplementary Methods for details.

### RNA Extraction and qRT‐PCR Analysis

Total cellular RNA was meticulously isolated utilizing the TRIzol reagent. Please see Supplementary Methods for details.

### Lentivirus and Plasmid Transfection

Small interfering RNAs (siRNAs) designed to target IER2, GMFG, and ITGB1, as well as inhibitors, were meticulously synthesized. Please see Supplementary Methods for details.

### Murine Xenograft Assays

Animal experiments were approved by the animal care ethics committees at the institute. Please see Supplementary Methods for details.

### Detection of Collagenous Fiber

Mason Trichrome and Picrosirius Red staining were used to detect collagenous fibers. Please see Supplementary Methods for details.

### Enzyme‐Linked Immunosorbent Assay (ELISA)

Plasma concentrations of GMFG, TGFB1, and PDGFA were evaluated using the Human GMFG ELISA Kit. Please see Supplementary Methods for details.

### Glutathione S‐Transferase (GST) Pull‐Down Assay

Please see Supplementary Methods for details.

### Sample Preparation and scRNA‐seq

Nine chordoma samples and five nucleus pulposus specimens were collected for scRNA‐seq. Single cell sequencing was performed as detailed in Supplementary Methods.

### Identification of Cell Type and Marker Genes

Clustering and visualization were performed using the R package Seurat 3.1 as detailed in Supplementary Methods.

### Identification of Different Expression Genes (DEGs) and Gene Enrichment

After annotating each cell type, the DEGs between specific clusters were identified, followed by enrichment analysis as detailed in Supplementary Methods.

### Sample Preparation and Visium Spatial Sequencing

Six fresh tumor specimens were selected for ST‐seq. Please see Supplementary Methods for details.

### Spatial Transcriptomics Expression Analysis

Seurat package was employed for conducting ST expression analysis, as detailed in Supplementary Methods.

### Digital Spatial Profiling (DSP)

A total of six tumor samples were specifically chosen for DSP using a GeoMx digital spatial profiler. Please see Supplementary Methods for details.

### Bulk RNA‐seq for U‐CH1 Cells

Total RNA was meticulously isolated from U‐CH1 cells that had been subjected to exogenous GMFG treatment or not using the TRIzol reagent. Please see Supplementary Methods for details.

### Data Independent Acquisition (DIA) Proteomic Analysis

Total protein content was extracted from supernatants of CAF before and after IER2 knockdown using the DB buffer. Please see Supplementary Methods for details.

### Gene Set Variation Analysis (GSVA)

Please see Supplementary Methods for details.

### Characterization of Immune Features in Chordoma

ESTIMATE and CIBERSORT were utilized to estimate immune infiltration. Please see Supplementary Methods for details.

### Evaluation of HALLMARK_HYPOXIA

The hypoxia‐related gene set HALLMARK_HYPOXIA (HALLMARK_HYPOXIA.v2023.2.Hs.gmt) was downloaded from the Gene Set Enrichment Analysis (GSEA) website (https://www.gsea‐msigdb.org/gsea/index.jsp). After this, the GSVA tool on the R platform was applied to perform enrichment analysis and scoring of the hypoxia‐related gene set using bulk RNA‐seq data from 126 tumor samples.

### Public Database

Two human chordoma bulk RNA‐seq datasets and 42 bone tumor cell lines RNA‐seq data in the Cancer Cell Line Encyclopedia were analyzed. Please see Supplementary Methods for details.

### Cell‐Cell Communication Analysis

CellChatDB was utilized to investigate cell‐cell communication. Please see Supplementary Methods for details.

### Molecular Docking Analysis

Interactively active components were selected based on their “Degree” and the most likely binding modes were predicted. Please see Supplementary Methods for details.

### Immunohistochemistry

Please see Supplementary Methods for details.

### Multiplexed Quantitative Immunofluorescence

Multiplexed Quantitative Immunofluorescence (QIF) staining was conducted on a total of 105 tumor samples. Please see Supplementary Methods for details.

### Spatial Distance Analysis

The HALO Next‐Generation Image Analysis software was used to evaluate the images. Please see Supplementary Methods for details.

### Statistical Analysis

Statistical analyses were executed using R version.

### Ethics Approval and Consent to Participate

The study protocol was approved by the Institutional Review Board at The First Affiliated Hospital, University of South China, Hunan, P.R. China. Written informed consent was obtained from each patient for publication of this study.

## Conflict of Interest

The authors declare no conflict of interest.

## Author Contributions

T.‐L.Z., B.‐W.Z., and C.X. are co‐first authors and contributed equally to this work. M.‐X.Z., W.H., and H.Z. are co‐corresponding authors and contributed equally to this work. All authors participated in data acquisition. M.‐X.Z., C.X., T.‐L.Z., and B.‐W.Z. contributed to the conception and design of the study. M.‐X.Z., C.X., P.‐F.W., B.‐W.Z., B.‐Y.Z., W.H., H.Z., and T.‐L.Z. did the data analysis and interpretation. M.‐X.Z., C.X., L.‐X.J., J.L., G.‐H.L., and T.‐L.Z. contributed to the drafting and revision of the manuscript. All authors read and approved the final manuscript.

## Supporting information

Supporting Information

Supporting Information

Supporting Information

## Data Availability

The data that support the findings of this study are available from the corresponding author upon reasonable request.
