# Supplementary material for: Hypoxic Upregulation of IER2 Increases Paracrine GMFG Signaling of Endoplasmic Reticulum Stress‐CAF to Promote Chordoma Progression via Targeting ITGB1
Source: Adv Sci (Weinh). 2024 Aug 29;11(40):2405421. doi: 10.1002/advs.202405421 (PMC11515918; doi:10.1002/advs.202405421)
Supplement: Supplementary file 1 — Supporting Information [file ADVS-11-2405421-s002.docx]

**Supplementary Methods**

***Patients and tissue samples***

This study was approved by the local ethical committee of our hospital and informed written consents were obtained from all patients. In this study, we included four independent cohorts. A total of 14 samples (including 9 chordoma and 5 nucleus pulposus [NP] tissues as controls) were included in the single-cell RNA sequencing (scRNA-seq) cohort. The baseline characteristics of scRNA-seq for the 9 patients are detailed in **Supplementary Table 1**. Six tumor samples were included in the ST cohort. For validation cohort, the QIF cohort comprised 105 patients (between June 2002 and December 2018) and was partially described in our previous studies^1^. Another cohort was composed of 126 patients from other institutes (between January 2002 and December 2019) and FFPE samples were used for bulk RNA-seq assay (RNA-seq cohort). All patients were confirmed to have classic chordoma, excluding other pathological subtypes including dedifferentiated or poorly differentiated tumors. Considering that prior tumor-specific interventions and comorbidities may likely distort the chordoma microenvironment, patients who had received any tumor-specific therapy (including radiotherapy, chemotherapy, or other targeted therapy) and those who had any immune-related diseases or other tumors on admission were excluded^1^.

Clinicopathologic characteristics were obtained from medical records and evaluated

as we previously detailed^1,2^. Fresh tumor specimens were also obtained during surgery for both single-cell RNA sequencing and spatial transcriptomics. The diagnosis was confirmed by two experienced pathologists based on the histological findings in hematoxylin and eosin (HE)-stained tissue sections, according to the previously described criteria^1,2^.

Followed-up data of patients were updated (until September 2020 for QIF cohort and June 2021 RNA-seq cohort) by performing regular clinical and radiographical examinations. The main outcome parameters of interest were local recurrence-free survival (LRFS), recorded as the length of time from the date of tumor resection to the first local recurrence, and overall survival (OS), defined as the time from surgery to death of any relative cause or until last follow-up. Observations were censored when patients were tumor-free (LRFS analysis) or alive (OS analysis).

***Cell lines***

The U-CH1 chordoma cell line (Catalogue Number: CRL-3217) was acquired from American Type Culture Collection (ATCC). Culture flasks were prepared following the guidelines provided by ATCC. A combination of Iscove's modified Dulbecco's medium (IMDM; ATCC, Catalogue Number: 30-2005) and RPMI-1640 medium (ATCC, Catalogue Number: 30-2001) in a ratio of 4:1 (v/v) was employed for cell cultivation. Similarly, the UM-Chor1 cell line was also obtained from the ATCC (Catalogue Number: CRL-3270). UM-Chor1 cells were cultured in IMDM with a 4:1 ratio of RPMI-1640. Both cell lines were supplemented with 10% FBS (Gibco, Catalogue Number: 10099141C) and 1% antibiotic mixture (Gibco, Catalogue Number: 15240062). Cells were grown in a humidified atmosphere at 37°C with 5% CO_2_.

***Isolation and culture of primary fibroblasts***

According to the method described previously with modifications^3^, CAFs and normal fibroblasts (NFs) were isolated from chordoma and adjacent normal tissues (>2 cm away from the tumors), respectively. In brief, tissues obtained from clinical chordoma specimens were meticulously sectioned into small fragments (<1 mm^3^) and then digested with 1 mg/mL collagenase Ⅰ at 37 °C for 1.5 h. Following this, the tissue fragments were immersed in PBS, and subsequently subjected to centrifugation at 800 rpm for 6 minutes. Concurrently, during the digestion process, a complete DMEM medium was introduced to terminate the enzymatic digestion. Thereafter, the cell suspension was distributed within a 6-well culture plate and left to adhere. Following a 2-day interval, the medium was replaced with a fresh culture medium. CAFs and NFs were routinely maintained in DMEM containing 10% FBS. The primary isolated CAFs were confirmed by immunofluorescence staining for α-SMA and Vimentin. All cell lines were kept at 37°C in an atmosphere of 5% CO_2_ and 95% air until subsequent use.

***Preparation of conditioned medium (CM)***

To obtain CAF- and NF-CM, primary CAFs and NFs were cultured for 48 h at 80%-90% density and the culture media were then collected and centrifuged at 3,000 rpm for 15 min to remove the cell debris. For treating the chordoma cells, the CM was added to the complete growth medium at a ratio of 1:2, and the cells were stimulated for 48 h at 37˚C^4^.

***Indirect co-culture***

CAFs were seeded in cell culture inserts (Transwell 0.4 μm pore size; Corning, Catalogue Number: 3422), whereas chordoma cells (1x10^3^ cells/cm^2^) were plated on the bottom of the wells and maintained in co-culture medium for 14 days. Then the chordoma cells were collected for subsequent analyses.

***Cell proliferation assay***

Cell growth was assessed using the Cell Counting Kit-8 (CCK8) assay^5^. Briefly, a total of 100 μL of fully supplemented IMDM medium along with 10 μL of CCK8 reagent (NCM Biotech, China, Catalogue Number: C6005) were introduced to the culture plates housing 8 × 10^3^ cells per well. The absorbance was measured at 450 nm. Data was systematically gathered over 4 consecutive days.

***Wound-healing assay***

The migratory ability of chordoma cells was evaluated using the Wound-healing assay^6^. In brief, chordoma cells were inoculated in six-well culture plates and allowed to settle overnight until 90% confluence. After serum starvation for 24 hours, a deliberate scratch was created to induce an artificial wound. The distance that cells had migrated was photographed by a digital camera (Leica Microsystems Inc., Bannockburn, IL, USA) under Inverted microscope (Olympus). Subsequently, the width of the wound was meticulously measured and subjected to detailed analysis.

***Transwell migration and invasion assays***

The invasion ability of chordoma cells was evaluated in transwell chamber (Corning, NY, USA, Catalogue Number: 3422) featuring pores measuring 8 micrometers^7^. In brief, 5×10^4^ chordoma cells in 300 μL of FBS-deprived IMDM medium were cultured in the upper chamber, and the lower plates were supplemented with 500 μL IMDM medium containing 0.1% FBS. Medium with 0.1% FBS served as a negative control and medium containing 10% FBS served as a positive control. The chambers were incubated for 48 hours. After removal of non-migrated cells on the upper layer of the membrane, cells that had migrated through the membrane were fixed in 4% paraformaldehyde and stained with 0.1% crystal violet. Following this, photomicrographs were captured by a microscope (Leica Microsystems Inc, Bannockburn, IL, USA). Subsequently, migratory cells were enumerated and subjected to comprehensive analysis.

***Detection of Reactive Oxygen Species (ROS) levels in CAFs via flow cytometry***

The cells were subjected to staining using the ROS kit (Solarbio, Bejing, China, Catalogue Number: CA1410) according to the provided manufacturer's instructions^8^. Subsequently, the cells were collected for assessment through flow cytometry. Expression data of superoxide dismutase (SOD), catalase (CAT), and malondialdehyde (MDA) were analyzed using FlowJo V.10 software.

***Western blotting***

Cells or tissues were washed with PBS and lysed using a RIPA lysis buffer containing 1% PMSF (Amresco, USA, Catalogue Number: 329-98-6) and 1% phosphatase inhibitor (Boster, Wuhan, China). After this, samples were centrifuged at 12,000 g for 10 min. BCA Protein Assay Kit (Beyotime Biotechnology, Shanghai, China, Catalogue Number: P0010S) was used to measure the protein concentration. An equal amount of protein was loaded onto a 10% SDS-PAGE gel (Epizyme Biomedical Technology, Shanghai, China, Catalogue Number: PG112) and transferred to PVDF membranes (Merk Millipore, Tulagreen, Carrigtwohill, Co Cork, IRELAND, Catalogue Number: IPVH00010). Membranes were blocked with 5% non-fat milk in PBST for 1 hr. Following this, the membranes were subjected to incubation with antibodies targeting IER2, ITGB1, GMFG, IRE1α, GRP78, XBP-1, CHOP, HIF-1α, PCNA, TGF-β, N-cadherin, E-cadherin, MMP2, YAP1, FAK, PI3K, p-PI3K, and GAPDH. Specific horseradish peroxidase (HRP)-conjugated secondary antibodies against IgG-mouse or rabbit were then applied to hybridize with the primary antibodies within a 37°C incubator. All Bands of western blots were quantified and normalized using Image J software. Comprehensive details regarding the antibodies are cataloged in the **Supplementary Table 2**

***RNA extraction and qRT-PCR analysis***

Total cellular RNA was meticulously isolated utilizing the TRIzol reagent (Invitrogen). Following extraction, the concentration of RNA samples was accurately determined via NanoDrop. Subsequently, complementary DNA (cDNA) synthesis was carried out using the isolated RNA in conjunction with the PrimeScript RT reagent Kit (TaKaRa, Japan). For quantitative real-time polymerase chain reaction (qRT-PCR), the SYBR Green Premix Ex Taq kit (Bio-Rad, CA, USA) was employed. Relative gene expression levels were quantified using the CFX96 Real-Time PCR Detection System (Bio-Rad, CA, USA). In this analysis, GAPDH served as the internal control. The complete list of primers employed for qPCR can be found in the **Supplementary Table 3**.

***Lentivirus, and plasmid transfection***

Small interfering RNAs (siRNAs) and short hairpin RNAs (shRNAs) designed to target IER2, GMFG, and ITGB1, as well as inhibitors, were meticulously synthesized. These si/shRNAs were incorporated into the construction of si/sh-IER2, GMFG, and ITGB1, or si/sh-NC lentiviral vectors. For boosting IER2 and ITGB1 expression, overexpression plasmids were acquired from Vigene Biosciences (Shandong, China). To ensure effective transfection, all cells were treated with lentivirus alongside 0.2% puromycin (Hanbio Biotechnology, Catalogue Number: HB-PU-500) for 72 hours. The transfection process was facilitated utilizing Opti-MEM (Gibco, Catalogue Number: 11058021) in conjunction with Lipofectamine 3000 (Invitrogen, Catalogue Number: L3000-015). Detailed sequences for interference, overexpression, and negative control can be found in the **Supplementary Table 3**.

***Murine xenograft assays***

Animal experiments were approved by the animal care ethics committees at our institute. C57BL/6J mice were procured from Vital River (Beijing, China) and were maintained within conditions that ensured their freedom from specific pathogens. Chordoma cells were mixed with an equal number of NFs or CAFs in 200 µL PBS at a 1:1 ratio and were subcutaneously injected into 4-week-old C57BL/6J mice. In the first experimental setup, CAFs subjected to Normoxia, Hypoxia, Hypoxia+anti-ERS drugs and U-CH1 cells were co-implanted subcutaneously into mice at a 1:1 ratio. In the second experimental setup, CAFs subjected to Hypoxia, Hypoxia+Lv-shNC, Hypoxia+Vector, Hypoxia+Lv-shIER2, Hypoxia+IER2 overexpression, Hypoxia+IER2 overexpression + anti-ERS drugs and U-CH1 cells were co-implanted subcutaneously into mice at a 1:1 ratio. The third experiment setup involved the co-implantation of CAFs treated with Normoxia, Normoxia+rGMFG, Hypoxia+Lv-sh-NC, Hypoxia+Lv-shGMFG, and U-CH1 cells subcutaneously into mice at a 1:1 ratio. In the last experiment setup, U-CH1 cells treated with NC, si-NC, si-ITGB1, si-ITGB1+rGMFG, and Hypoxia treated-CAFs were co-transplanted subcutaneously into mice at a ratio of 1:1. Additionally, exogenous GMFG was simultaneously injected locally. Tumor measurements were taken five times every 3 days using Vernier calipers to gauge both the long diameter (I) and short diameter (W) of the tumors. Tumor volume was calculated using the formula 0.52×(I×W^2^). On day 28, the mice were humanely euthanized, and the tumors were extracted and weighed.

***Detection of collagenous fiber***

Mason Trichrome and Picrosirius Red staining were used to detect collagenous fibers. A portion of the tumor extracted from mice was cut and washed with PBS. Then the tissue was fixed and embedded using the 4% paraform. The 5-μm thick slice was prepared for subsequent staining. In Masson’s trichrome staining, the collagen fibrils appeared blue^9^. The Picrosirius Red stain was performed to detect the distributions of each type of collagenous fibers^10^.

***Enzyme-linked immunosorbent assay (ELISA)***

Plasma concentrations of GMFG, TGFB1, and PDGFA were evaluated using the Human GMFG ELISA Kit (abbexa, Catalogue Number: abx387591), Human TGFB1 ELISA Kit (Abcam, Catalogue Number: ab100647), and Human PDGFA ELISA Kit (Abcam, Catalogue Number: ab100622), respectively. This was executed following the procedural guidelines provided by the manufacturer. The absorbance (450 nm) of all sample was detected on a standard automatic microplate reader.

***Glutathione S-transferase (GST) pull-down assay***

The procedure for the GST pull-down assay was previously documented^11^. In brief, GST-GMFG fusion proteins were induced and purified from E. coli according to the manufacturer's instruction. These fusion proteins were then reconstituted in PBS supplemented with protease inhibitors and 0.5% Nonidet P-40. Approximately 100 µg of GST and GST-GMFG fusion protein, tethered to beads, were subjected to incubation with 1 ml of cell lysates under gentle rotation. Subsequently, the proteins were eluted from the beads using 2x SDS-PAGE sample buffer. The GST-GMFG fusion protein was incubated with ITGB1, and eventually identified through western blotting technique.

***Sample preparation and scRNA-seq***

The scRNA-seq for chordoma tissues was performed as we previously detailed^12^. In brief, the freshly resected samples were dissected into <1mm^3^ fragments for subsequent examinations and rinsed in phosphate-buffered saline (PBS). Then the tumor specimens were digested using 0.1% trypsin (Corning Inc, New York), and 0.5 mg/mL collagenase type IV (Thermo Fisher Scientific Inc.), resulting in the isolation of individual cells. After treated with red blood cell (RBC) lysis buffer (Invitrogen, Waltham, Massachusetts), the dissociated cells were stained using 0.4% trypan blue (Solarbio, Beijing, China) to assess cell viability. Subsequently, the single-cell suspension was loaded onto the 10 × Genomics single-cell-A chip to enable the capture of 3000 cells per chip position according to the established procedure. The cDNA library construction was performed following the customary instructions from the 10 × Genomics Single Cell 3′ v2 Reagent Kit (Pleasanton, California). This library was then subjected to sequencing using a HiSeq 2500 instrument, facilitated by Novogene Bio Technology Inc (Tianjin, China). To handle the raw sequencing data, the Cell Ranger software (version 3.0.2) was employed. The generation of gene-barcode matrices occurred, which were subsequently imported into the Seurat software (version 2.3.4), an R package for analyses. After normalization using the Normalize Data function within Seurat, the gene expression matrices originating from 14 distinct samples (including 9 chordoma and 5 NP tissue specimens as controls) were integrated to ensure accurate analysis and these data have been partially communicated by our group^11^. To mitigate batch effects, a combination of canonical correlation analysis and mutual nearest neighbors-anchors was adopted utilizing the Seurat package.

***Identification of cell type and marker genes***

Clustering and data visualization of scRNA-seq profile were expertly executed utilizing the R package Seurat 3.1. Briefly, the FindVariableGenes function was employed to pinpoint the top 2000 genes with the highest variability. This gene set was then subjected to Principal Component Analysis (PCA) through the implementation of the RunPCA function. This analysis was conducted using the top 20 significant principal components. These clusters were then dimensionality-reduced and visually presented using the RunUMAP function. No artificial selection or intervention was used during the process. In addition, we manually removed the doublets in cell clustering. We characterize different cell types by referring to the expression profiles of established typical markers in the Cell Marker database (http://xteam.xbio.top/CellMarker/). The FindMarkers function was utilized o identify the specific marker genes for each cluster and subgroup.

***Identification of different expression genes (DEGs) and gene enrichment***

The FindMarkers function was used to pinpoint the DEGs for each cluster. These DEGs were subsequently refined based on specific criteria: |log2fold change| > 0.5 and adjusted FDR < 0.05 through the Bonferroni method. The R package clusterProfiler function was also used to execute an enrichment analysis involving Gene Ontology terms, which unraveled the functional implications of the identified DEGs.

***Sample preparation and Visium Spatial Sequencing***

Visium Spatial transcriptomics sequencing (ST-seq) was performed for chordoma tissues as we previously described^12^. In brief, six fresh tumor specimens were selected for ST-seq and three samples have been previously described in our study^12^. The Visium Spatial Tissue Optimization Slide & Reagent kit (10 × Genomics, Pleasanton, California) was used to optimize the conditions of permeabilization for tumor samples according to the User Guide (CG000238, 10 × Genomics) before sequencing. The optimal time of permeabilization was 15 min. Using the Visium Spatial Gene Expression Slide & Reagent kit (10 × Genomics), the ST cDNA libraries were prepared and then sequenced using a Novaseq PE150 platform (Illumina, San Diego, California) with a sequencing depth of average 250 million read-pairs per sample as per the User Guide (CG000239, 10 × Genomics).

***Spatial transcriptomics expression analysis***

Analysis of the ST-seq data was performed as we previously documented^12^. Briefly, with the Space Ranger v1.2.0 (10 × Genomics) software, raw FASTQ files and histology images were aligned with STAR v.2.5.1b to Human GRCh38 reference genome. The Seurat package was used to perform the ST expression analysis. The spot matrix was filtered out to retain only the spots with more than 100 detected genes. Normalization for individual count matrices was conducted with the regularized negative binomial regression (SCTransform) function. For dimensionality reduction, 3000 genes with the most variable expression levels were screened using the FindVariableGenes function and selected for principal component analysis (PCA). Using the FindClusters function, the first 20 principal components were used for spot clustering. Visualization of ST clusters was carried out using the uniform manifold approximation and projection (UMAP). The DEGs for each cluster were screened via FindAllMarkers function. Genes with fold change larger than 2 and adjusted P-value < 0.05 were retrieved. Co-expression analysis of target genes was performed using the Seurat package^13^.

***Digital spatial profiling (DSP)***

A total of six tumor samples were specifically chosen for DSP using a GeoMx digital spatial profiler (NanoString Technologies, Seattle, WA). The detailed procedures were described elsewhere^14^. Briefly, formalin-fixed paraffin-embedded (FFPE) sections underwent the processes of deparaffinization and rehydration, followed by overnight incubation with a trio of fluorescence-labeled visualization antibodies (including PanCK [GeoMx Solid Tumor Morp Kit HsR, NanoString Technologies, Catalogue Number: 121300310], CD68 [Zsbio, Catalogue Number: ZM0060] and α-SMA [Abcam, Catalogue Number: ab202296] ), aimed at detecting markers-related to macrophages, tumoral and stromal cells. After staining, digital fluorescent images of the tissue samples were captured using the GeoMx DSP instrument (NanoString Technologies). Afterwards, discrete regions of interest (ROI) were generated and partitioned into two distinct tissue compartments with well-defined molecular attributes: the tumoral and stromal regions. This process was facilitated through the utilization of UV-guided technology. Oligos extracted from these ROIs were then released and accumulated within 96-well microtiter plates. Subsequently, they were hybridized to optical barcodes and enumerated within the nCounter system (NanoString Technologies) to measure mRNA expression. The resulting digital count datasets underwent an initial normalization process utilizing internal spike-in controls (ERCCs) and were subsequently adjusted relative to their respective compartment's area. For the sake of data quality, compartments characterized by nuclei counts fewer than 10 or an area of illumination less than 100 μm² were excluded from the analyses.

***Bulk RNA-seq for U-CH1 cells***

Total RNA was meticulously isolated from U-CH1 cells that had been subjected to exogenous GMFG treatment or not using the TRIzol reagent (Invitrogen, Catalogue Number: 15596018). Before RNA extraction, the cells were exposed to 3 μM cisplatin for 24 hours. NanoDrop was employed to determine the RNA concentration, while the Agilent Bioanalyzer 2100 (Agilent Technologies, CA, USA) was utilized to gauge dsRNA integrity. Subsequently, the transcriptomic sequencing libraries were prepared using the VAHTS Stranded mRNA-seq Library Prep Kit (Illumina). The library concentration was quantified via a Qubit^®^ 2.0 Fluorometer (Life Technologies, CA, USA) and quantitative PCR. The insert size was assessed using the Agilent Bioanalyzer 2100 system. After rigorous quality assessment, the library underwent sequencing on the Illumina Novaseq PE150 platform. To ensure the high-quality clean reads, those containing poly-N, adaptors, and those of low quality were filtered out using FastQ Screen (<https://www.bioinformatics.babraham.ac.uk/projects/fastq_screen/>). For quantifying gene expression levels, high-throughput sequencing analysis was applied. DEGs were then identified via edgeR (with criteria set at |log2FoldChange|>0.4 and an adjusted p-value <0.05).

***Data independent acquisition (DIA) proteomic analysis***

Total protein content was extracted from supernatants of CAF before and after IER2 knockdown using the DB buffer. For quality inspection, 20μg protein underwent determination of concentration before being subjected to sodium dodecyl sulfate-polyacrylamide gel electrophoresis (SDS-PAGE). The extracted proteins were then digested with trypsin (Promega/V5280) and the peptide mixture was collected. 3μg peptide dissolved with bufer A (0.1% formic acid) was fractionated and collected with Vanquish™ Neo UHPLC system (Termo Fisher scientifc, MA, USA), followed by mass spectrometry analysis conducted by Thermo orbitrap astral mass spectrometer (Termo Fisher scientifc, MA, USA). Raw data were analyzed using DIA-NN (Thermo Fisher, San Jose, CA, USA) with specific parameters, which included static modification involving alkylation modification of cysteine, dynamic modifications encompassing oxidation (M) and acetylation (N-terminal), and trypsin digestion settings. Peptides with FDR > 1% were filtered. Differentially expressed proteins (DEPs) were analyzed the R software (v 3.2.1). A *P*-value < 0.05 and |log2fold change (FC)| > 0.58 were set as the criteria for DEPs screening. Gene Ontology (GO) term and Kyoto Encyclopedia of Genes and Genomes (KEGG) pathway analyses were performed to categorize the DEPs.

***Gene set variation analysis (GSVA)***

We obtained relevant gene sets from the MSigDB database (https://www.gsea-msigdb.org/) and calculated the signature score of corresponding gene set using GSVA^12^.

***Characterization of immune features in chordoma***

We utilized two methods, ESTIMATE and CIBERSORT, to estimate immune infiltration. ESTIMATE was employed through the ESTIMATE R package to evaluate the overall immune infiltration (ESTIMATEScore) and ImmuneScore based on the analyses of bulk RNA-seq data from 126 tumor samples. Similarly, CIBERSORT (http://cibersort.stanford.edu/) was utilized for quantifying the abundance of 22 types of immune cells in chordoma microenvironment^15^.

***Public database***

We analyzed two human bulk RNA-seq datasets (GSE239531 and GSE205457), comprising 20 and 6 chordoma samples, respectively. Additionally, we included RNA-seq data from 42 bone tumor cell lines publicly available in the Cancer Cell Line Encyclopedia (DepMap ID: ACH-000039, ACH-000041, ACH-000052, ACH-000082, ACH-000087, ACH-000210, ACH-000279, ACH-000359, ACH-000364, ACH-000391, ACH-000410, ACH-000418, ACH-000424, ACH-000499, ACH-000516, ACH-000613, ACH-000748, ACH-001001, ACH-001022, ACH-001029, ACH-001030, ACH-001032, ACH-001034, ACH-001035, ACH-001038, ACH-001192, ACH-001193, ACH-001283, ACH-001430, ACH-001431, ACH-001519, ACH-001526, ACH-001712, ACH-001715, ACH-001814, ACH-001818, ACH-002067, ACH-002069, ACH-002471, ACH-002780, ACH-002781, ACH-002834), obtained from the DepMap portal (https://depmap.org/portal/). All datasets were downloaded from publicly available databases, and data collection and usage comply with the publication guidelines and data access policies of the respective databases.

***Cell-cell communication analysis***

To explore potential interactions between cells, we utilized CellChatDB to investigate cell-cell communication based on ligands, receptors, and their interactions. We extracted significant ligand-receptor pairs based on permutation tests computed with a p-value < 0.05. The R packages igraph and ggplot2 were employed for visualizing the results.

***Molecular docking analysis***

We selected interactively active components based on their "Degree" and predicted the most likely binding modes between active ingredients and potential targets^16^. For molecular docking analysis, we utilized AutoDockTools 1.5.7 and PyMOL software (version 2.1, USA) for computation and visualization. The 3D structure of the target proteins was retrieved from the Protein Data Bank (PDB, https://www.rcsb.org/) database.

***Immunohistochemistry***

Following our previous description^17^, immunohistochemical staining was performed. In brief, paraffin-embedded sections (4μm) of tumor specimens from four institutes were deparaffinized in xylene, rehydrated through a series of graded ethanol solutions, and then rinsed in distilled water. Subsequently, after antigen retrieval and blocking, tissue sections were incubated overnight at 4°C with primary antibodies **(Supplementary Table 2).** Following incubation with biotinylated secondary antibodies (goat anti-rabbit or anti-mouse immunoglobulins), immunodetection was performed using the avidin-biotin-peroxidase complex, and then visualized with 3,3'-diaminobenzidine solution and counterstained with hematoxylin. The IHC H-score system was employed to score the stained sections. The final value is the product of the staining intensity (scored from 0 to 3) and the percentage of stained cells (ranging from 0 to 100)^18^.

***Multiplexed quantitative immunofluorescence***

Multiplexed Quantitative Immunofluorescence (QIF) staining was conducted on a total of 105 tumor samples. This process was conducted using the Opal 7-color Manual Immunohistochemistry (IHC) Kit from PerkinElmer (Waltham, Massachusetts) according to the established methodology^12^. The analyzed cohort comprised 31 female and 74 male patients. Within this cohort, 92 cases were spinal chordomas, while 13 belonged to cranial chordomas. All tumors were verified as the conventional pathology type. The average follow-up period spanned 42.10 ± 35.18 months, and no patients were lost to follow-up.

In brief, each tumor tissue section underwent concurrent staining with isotype-specific primary antibodies, which enabled the detection of multiple cellular components, encompassing the tumor compartment (marked by CK19^+^ cells), total CAFs (marked by α-SMA^+^ cells), and ERS-CAF (marked by α-SMA^+^DNAJB1^+^HSPA1A^+^ cells), as well as the expression level of stromal IER2 and tumoral ITGB1. To further corroborate the interactive SPP1/ITGB1signaling between TAMs and tumor cells, we performed the QIF assay using the same method described above. Isotype-specific primary antibodies were used to detect SPP1^+^TAMs (specifically CD68^+^SPP1^+^ TAMs). Nuclei were stained with 4’,6-diamidino-2-phenylindole (DAPI) and the tumor mask was delineated through CK19, as recommended in prior studies^12^. Specifically, formalin-fixed paraffin-embedded (FFPE) tumor sections underwent deparaffinization, followed by antigen retrieval in a pressure cooker with Tris-ethylenediaminetetraacetic acid buffer (pH 9.0) for 10 minutes. Subsequently, sections were subjected to primary antibody incubation at 4°C overnight, post-antigen blocking using 3% H_2_O_2_ for 15 minutes and 10% goat serum for 30 minutes at room temperature. Horseradish peroxidase (HRP)-conjugated secondary antibodies were then incubated for 1 hour at room temperature, followed by tyramide-based HRP activation lasting 20 minutes at 37°C. The residual HRP activation was quenched with 1 mM benzoic hydrazide supplemented with 0.15% H_2_O_2_. Detection of CK19, α-SMA, HSPA1A, DNAJB1, IER2, and ITGB1 was achieved using XTSA 780, XTSA 480, XTSA 690, XTSA 620, XTSA 520, and Opal 570 conjugates, respectively. Similarly, detection of CK19, ITGB1,CD68, and SPP1 was achieved using XTSA 480, XTSA 520, XTSA 780, and Opal 620 conjugates, respectively. Finally, coverslips were applied using ProLongGold Antifade reagent containing DAPI, and the slides were allowed to dry overnight. Details of the antibodies utilized can be found in **Supplementary Table 4**.

***Spatial distance analysis***

To assess spatial distance between target cell subpopulations, the HALO Next-Generation Image Analysis software (version 2.1.1637.18, Indica Labs, Albuquerque, NM) was used to evaluate the images according to the previously documented procedures^12^. In brief, the entire tumor section images were subjected to scanning using a Vectra system (version 2.0.8, PerkinElmer) with a 4x objective. This scanning procedure was standardized with uniform laser power, bit depth, and exposure time to ensure consistent comparability. The acquired QIF images were subsequently imported into the HALO software for detailed analysis. Images displaying staining artifacts or encompassing less than 3% tumor tissue were excluded. Cell nuclei were identified utilizing DAPI staining, and the tumor mask was established via CK19 staining. Within the DAPI compartment, the stroma compartment was defined, excluding the tumor subregion.

To facilitate quantification, all nuclei within the QIF image were automatically segmented using the commercially available High-Plex FL module (version 2.0). Quantitative assessments of distinct cell subsets were calculated by dividing the count of positive cells by the area of the entire tissue section. The derived data was expressed as positive cells per million (10^6^) pixels^2^. A marker was classified as positive if its QIF score surpassed the signal detection threshold, specifically set at 800 for CK19, 900 for αSMA, 1700 for ITGB1, 350 for DNAJB1, 700 for HSPA1A, 400 for IER2, 370 for CD68, and 950 for SPP1. These threshold values were determined based on the overall expression patterns of CAF throughout the complete tissue section, along with visual inspection^12^.

For the analysis of cell-to-cell distances, the spatial coordinates of IER2^+^ or IER2^-^ ERS-CAF and ITGB1^+^ tumor cells were imported into a spatial plot within the HALO software. Leveraging this spatial plot, the spatial analysis algorithm was exploited to quantify the distance between IER2^+/-^ ERS-CAF subtypes and their nearest tumor cells. Furthermore, the effective percentage of IER2^+/-^ ERS-CAFs within the range of 0 to 100 μm radii of ITGB1^+^ tumor cells was calculated in consecutive increments of 10 μm. This effective percentage denoted the proportion of IER2^+/-^ ERS-CAF density within the specified radii of an ITGB1^+^ tumor cell relative to the total number of corresponding IER2^+/-^ ERS-CAFs present within the entire tumor section^12^. Ultimately, the averages for distance and effective percentage data were computed for subsequent analytical procedures^12^. Similarly, we compared the expression level of SPP1 on TAMs, as well as ITGB1 on tumor cells according to the consecutively increasing 10 μm distance up to 100 μm between TAMs and tumor cells.

***Statistical analysis***

Continuous data were succinctly summarized through the presentation of mean values accompanied by their respective standard deviations. For comprehensive data scrutiny, a combination of statistical techniques was employed: Student's t-test and the One-Way ANOVA test. On the other hand, categorical data were aptly documented in terms of frequency or composition ratios. Subsequent analysis of these categorical variables was facilitated through the application of the chi-square test. To delve into potential relationships between continuous variables, Pearson's correlation test was utilized. For survival analysis involving continuous data, a specialized tool known as the Cutoff Finder Web Application (https://molpathoheidelberg.shinyapps.io/CutoffFinder_v1/) was harnessed to determine the cutoff point with OS as the outcome parameter^2^. This tool provided the requisite threshold value for survival analysis. More specifically, the threshold value was identified as the point marked by the lowest corrected *P*-value obtained from the log-rank test^19^. Visualization of survival outcomes was undertaken through the application of the Kaplan-Meier method, which effectively produced the LRFS and OS curves. These curves were then subjected to survival rate comparison within subgroups, utilizing the log-rank test to uncover any differences. All statistical analyses were executed using R (R Foundation for Statistical Computing, Vienna, Austria). Following standard practice, all statistical tests were two-sided. Statistically significant disparities were defined by a *P*-value of less than 0.05. Significance levels were denoted as follows: * or **^#^** for *P* < 0.05, ** or **^##^** for *P* < 0.01, *** or **^###^** for *P* < 0.001, and **** or **^####^** for *P* < 0.0001.

**References**

1. Xia C, Huang W, Chen YL*, et al.* Coexpression of HHLA2 and PD-L1 on Tumor Cells Independently Predicts the Survival of Spinal Chordoma Patients. *Front Immunol* 2022; 12: 797407.

2. Zou MX, Pan Y, Huang W*, et al.* A four-factor immune risk score signature predicts the clinical outcome of patients with spinal chordoma. *Clin Transl Med* 2020; 10: 224-37.

3. Yang Y, Ma Y, Yan S*, et al.* CAF promotes chemoresistance through NRP2 in gastric cancer. *Gastric Cancer* 2022; 25: 503-14.

4. Sung JS, Kang CW, Kang S*, et al.* ITGB4-mediated metabolic reprogramming of cancer-associated fibroblasts. *Oncogene* 2020; 39: 664-76.

5. Chen X, Liu X, Li QH*, et al.* A patient-derived organoid-based study identified an ASO targeting SNORD14E for endometrial cancer through reducing aberrant FOXM1 Expression and β-catenin nuclear accumulation. *J Exp Clin Cancer Res* 2023; 42: 230.

6. Hu QR, Huang QX, Hong H*, et al.* Ginsenoside Rh2 and its octyl ester derivative inhibited invasion and metastasis of hepatocellular carcinoma via the c-Jun/COX2/PGE2 pathway. *Phytomedicine* 2023; 121: 155131.

7. Zhang Y, Zhao G, Yu L*, et al.* Heat-shock protein 90α protects NME1 against degradation and suppresses metastasis of breast cancer. *Br J Cancer* 2023; 129: 1679-91.

8. Wang X, Zhou Y, Ning L*, et al.* Knockdown of ANXA10 induces ferroptosis by inhibiting autophagy-mediated TFRC degradation in colorectal cancer. *Cell Death Dis* 2023; 14: 588.

9. Chen Z, Han X, Ouyang X*, et al.* Transplantation of induced pluripotent stem cell-derived mesenchymal stem cells improved erectile dysfunction induced by cavernous nerve injury. *Theranostics* 2019; 9: 6354-68.

10. Chen PJ, Dutra EH, Mehta S*, et al.* Age-related changes in the cartilage of the temporomandibular joint. *Geroscience* 2020; 42: 995-1004.

11. Zhang Z, Sun D, Tang H*, et al.* PER2 binding to HSP90 enhances immune response against oral squamous cell carcinoma by inhibiting IKK/NF-κB pathway and PD-L1 expression. *J Immunother Cancer* 2023; 11.

12. Zhang TL, Xia C, Zheng BW*, et al.* Integrating single-cell and spatial transcriptomics reveals endoplasmic reticulum stress-related CAF subpopulations associated with chordoma progression. *Neuro Oncol* 2024; 26: 295-308.

13. She C, Wu C, Guo W, et al. Combination of RUNX1 inhibitor and gemcitabine mitigates chemo-resistance in pancreatic ductal adenocarcinoma by modulating BiP/PERK/eIF2α-axis-mediated endoplasmic reticulum stress. *J Exp Clin Cancer Res*. 2023; 42: 238.

14. Merritt CR, Ong GT, Church SE*, et al.* Multiplex digital spatial profiling of proteins and RNA in fixed tissue. *Nat Biotechnol* 2020; 38: 586-99.

15. Han S, Wang Q, Shen M*, et al.* Immunogenic cell death related mRNAs associated signature to predict immunotherapeutic response in osteosarcoma. *Heliyon* 2024; 10: e27630.

16. Liu C, Hu W, Feng X*, et al.* Network pharmacology analysis of a patented Chinese herbal medicine for alleviating anxiety disorder in in vitro fertilization-embryo transfer. *J Tradit Complement Med* 2024; 14: 191-202.

17. Zheng BW, Zheng BY, Niu HQ*, et al.* Tumor Growth Rate in Spinal Giant Cell Tumors of Bone and Association With the Immune Microenvironment and Denosumab Treatment Responsiveness: A Multicenter Study. *Neurosurgery* 2023; 92: 524-37.

18. Klümper N, Ralser DJ, Ellinger J*, et al.* Membranous NECTIN-4 Expression Frequently Decreases during Metastatic Spread of Urothelial Carcinoma and Is Associated with Enfortumab Vedotin Resistance. *Clin Cancer Res* 2023; 29: 1496-505.

19. Altman DG, Lausen B, Sauerbrei W*, et al.* Dangers of using "optimal" cutpoints in the evaluation of prognostic factors. *J Natl Cancer Inst* 1994; 86: 829-35.
