# Supplementary material for: Hypoxic Upregulation of IER2 Increases Paracrine GMFG Signaling of Endoplasmic Reticulum Stress‐CAF to Promote Chordoma Progression via Targeting ITGB1
Source: Adv Sci (Weinh). 2024 Aug 29;11(40):2405421. doi: 10.1002/advs.202405421 (PMC11515918; doi:10.1002/advs.202405421)
Supplement: Supplementary file 2 — Supporting Information [file ADVS-11-2405421-s003.docx]

**Supplementary Table 1.** Baseline characteristic of 14 patients for scRNA-seq

| Number | Characteristic | | |
| --- | --- | --- | --- |
|  | Age (years) | Sex | Location |
| CH 1 | 60 | Male | Sacral vertebra |
| CH 2 | 53 | Male | Sacral vertebra |
| CH 3 | 86 | Female | Sacral vertebra |
| CH 4 | 48 | Male | Sacral vertebra |
| CH 5 | 65 | Male | Sacral vertebra |
| CH 6 | 78 | Female | Sacral vertebra |
| CH 7 | 70 | Female | Sacral vertebra |
| CH 8 | 59 | Male | Sacral vertebra |
| CH 9 | 66 | Female | Sacral vertebra |
| NP 1 | 51 | Female | Lumbar vertebra |
| NP 2 | 47 | Female | Lumbar vertebra |
| NP 3 | 35 | Male | Lumbar vertebra |
| NP 4 | 60 | Female | Lumbar vertebra |
| NP 5 | 34 | Male | Lumbar vertebra |

Abbreviations: CH, chordoma; NP, nucleus pulposus.

**Supplementary Table 2****.** Detailed information on the antibodies used for the western blot and immunohistochemistry

| Antibody | Host Species | Description | Catalog Number | Company | Dilution |
| --- | --- | --- | --- | --- | --- |
| GAPDH | Mouse | Monoclonal | 60004-1-IG | Proteintech | 1:50000 |
| HIF-1α | Rabbit | Polyclonal | 20960-1-AP | Proteintech | 1:2000 |
| IER2 | Rabbit | Polyclonal | 23849-1-AP | Proteintech | 1:100 |
| IRE1α | Rabbit | Polyclonal | 27528-1-AP | Proteintech | 1:1000 |
| GRP78 | Rabbit | Polyclonal | 11587-1-AP | Proteintech | 1:2000 |
| XBP-1 | Rabbit | Polyclonal | 24168-1-AP | Proteintech | 1:1000 |
| CHOP | Rabbit | Polyclonal | 15204-1-AP | Proteintech | 1:500 |
| PCNA | Rabbit | Polyclonal | 10205-2-AP | Proteintech | 1:5000 |
| TGF-β1 | Rabbit | Polyclonal | 21898-1-AP | Proteintech | 1:1000 |
| E-cadherin | Rabbit | Polyclonal | 20874-1-AP | Proteintech | 1:20000 |
| N-cadherin | Rabbit | Polyclonal | AF4039 | Affinity | 1:500 |
| MMP-2 | Rabbit | Polyclonal | CSB-PA003258 | CUSABIO | 1:500 |
| ITGB1 | Rabbit | Polyclonal | CSB-PA011266 | CUSABIO | 1:500 |
| GMFG | Rabbit | Polyclonal | DF12405 | Affinity | 1:500 |
| YAP1 | Rabbit | Polyclonal | 13584-1-AP | Proteintech | 1:2000 |
| FAK | Rabbit | Polyclonal | 12636-1-AP | Proteintech | 1:500 |
| PI3K | Mouse | Monoclonal | 60225-1-Ig | Proteintech | 1:500 |
| p-PI3K | Rabbit | Polyclonal | AF3242 | Affinity | 1:500 |
| GAPDH | Rabbit | Polyclonal | AF7021 | Affinity | 1:3000 |

**Supplementary Table 3.** Detailed information on the primers used for the q-PCR and transfection

| Gene Name | Primer（5'-3'） |
| --- | --- |
| HIF1α | GAACGTCGAAAAGAAAAGTCTCG |
|  | CCTTATCAAGATGCGAACTCACA |
| IRE1α | AGAGAAGCAGCAGACTTTGTC |
|  | GTTTTGGTGTCGTACATGGTGA |
| GRP78 | GAAAGAAGGTTACCCATGCAGT |
|  | CAGGCCATAAGCAATAGCAGC |
| XBP-1 | CCCTCCAGAACATCTCCCCAT |
|  | ACATGACTGGGTCCAAGTTGT |
| CHOP | GAACGGCTCAAGCAGGAAATC |
|  | TTCACCATTCGGTCAATCAGAG |
| PCNA | CCTGCTGGGATATTAGCTCCA |
|  | CAGCGGTAGGTGTCGAAGC |
| TGF-β | GGCCAGATCCTGTCCAAGC |
|  | GTGGGTTTCCACCATTAGCAC |
| E-Caderhin | ATTTTTCCCTCGACACCCGAT |
|  | TCCCAGGCGTAGACCAAGA |
| N-Caderhin | TCAGGCGTCTGTAGAGGCTT |
|  | ATGCACATCCTTCGATAAGACTG |
| MMP2 | CCCACTGCGGTTTTCTCGAAT |
|  | CAAAGGGGTATCCATCGCCAT |
| IER2 | ACTGGTCCCGAGCAAGAAAG |
|  | CGACTTCGGATGACGCTCC |
| FAK | AGTGGACCAGGAAATTGCTTTG |
|  | GTGTTTTGGCCTTGACAGAATC |
| YAP1 | TAGCCCTGCGTAGCCAGTTA |
|  | TCATGCTTAGTCCACTGTCTGT |
| ITGB1 | CCTACTTCTGCACGATGTGATG |
|  | CCTTTGCTACGGTTGGTTACATT |
| GAPDH | ACAGCCTCAAGATCATCAGC |
|  | GGTCATGAGTCCTTCCACGAT |
| si-NC | UUCUCCGAACGUGUCACGUTT |
|  | ACGUGACACGUUCGGAGAATT |
| si-IER2 | CGGCGGACAGCAUGCUCAATT |
|  | UUGAGCAUGCUGUCCGCCGTT |
| sh-IER2 | GATCCCGGCGGACAGCATGCTCAACTCGAGTTGAGCATGCTGTCCGCCGTTTTTG |
|  | AATTCAAAAACGGCGGACAGCATGCTCAACTCGAGTTGAGCATGCTGTCCGCCGG |
| IER2 oe | CTAGCGTTTAAACTTAAGCTTATGGAAGTGCAGAAAGAGGCAC |
|  | TGCTGGATATCTGCAGAATTCTCAGAAGGCCACCACGGC |
| Lv-IER2 | CTACCGGACTCAGATCTCGAGATGGAAGTGCAGAAAGAGGCAC |
|  | GTACCGTCGACTGCAGAATTCTCAGAAGGCCACCACGGC |
| si-GMFG | GGUUCGUGGUUUACAGCUATT |
|  | UAGCUGUAAACCACGAACCTT |
| sh-GMFG | GATCCGGTTCGTGGTTTACAGCTACTCGAGTAGCTGTAAACCACGAACCTTTTTG |
|  | AATTCAAAAAGGTTCGTGGTTTACAGCTACTCGAGTAGCTGTAAACCACGAACCG |
| GMFG-oe | CTAGCGTTTAAACTTAAGCTTATGTCTGACTCCCTGGTGGTGT |
|  | TGCTGGATATCTGCAGAATTCTCAACGAAAGAAAGACAACTTTTCTT |
| Lv-GMFG | CTACCGGACTCAGATCTCGAGATGTCTGACTCCCTGGTGGTGT |
|  | GTACCGTCGACTGCAGAATTCTCAACGAAAGAAAGACAACTTTTCTT |
| si-ITGB1 | GCAAAUUCUAGCAAUGUAATT |
|  | UUACAUUGCUAGAAUUUGCTT |
| sh-ITGB1 | GATCCGCAAATTCTAGCAATGTAACTCGAGTTACATTGCTAGAATTTGCTTTTTG |
|  | AATTCAAAAAGCAAATTCTAGCAATGTAACTCGAGTTACATTGCTAGAATTTGCG |
| ITGB1-oe | CTAGCGTTTAAACTTAAGCTTATGAATTTACAACCAATTTTCTGGAT |
|  | TGCTGGATATCTGCAGAATTCTCATTTTCCCTCATACTTCGGATT |
| Lv-ITGB1 | CTACCGGACTCAGATCTCGAGATGAATTTACAACCAATTTTCTGGAT |
|  | GTACCGTCGACTGCAGAATTCTCATTTTCCCTCATACTTCGGATT |

**Supplementary Table 4.** Detailed information on the antibodies used for the quantitative immunofluorescence assay

| Antibody | Host Species | Description | Catalog Number | Company | Dilution |
| --- | --- | --- | --- | --- | --- |
| α-SMA | Mouse | Monoclonal | ab7817 | Abcam | 1:1000 |
| Vimentin | Rabbit | Monoclonal | 10366-1-AP | Proteintech | 1:300 |
| SPP1 | Rabbit | Monoclonal | Ab214050 | Abcam | 1:2000 |
| CK19 | Rabbit | Monoclonal | Ab52625 | Abcam | 1:800 |
| ITGB1 | Rabbit | Monoclonal | 34971 | CST | 1:100 |
| CD68 | Mouse | Monoclonal | ZM0060 | [Zsbio](http://www.zsbio.com/aboutus/briefintroduce) | 1:500 |
| IER2 | Rabbit | Monoclonal | 23849-1-AP | Proteintech | 1:200 |
| HSPA1A | Mouse | Monoclonal | ab5439 | Abcam | 1:2000 |
| DNAJB1 | Mouse | Monoclonal | TA502194 | OriGene | 1:200 |
