# Supplementary material for: Hypoxic Upregulation of IER2 Increases Paracrine GMFG Signaling of Endoplasmic Reticulum Stress‐CAF to Promote Chordoma Progression via Targeting ITGB1
Source: Adv Sci (Weinh). 2024 Aug 29;11(40):2405421. doi: 10.1002/advs.202405421 (PMC11515918; doi:10.1002/advs.202405421)
Supplement: Supplementary file 3 — Supporting Information [file ADVS-11-2405421-s001.pdf]

ADVANCED SCIENCE
Open Access

Supporting Information

for *Adv. Sci.*, DOI 10.1002/advs.202405421

Hypoxic Upregulation of IER2 Increases Paracrine GMFG Signaling of Endoplasmic Reticulum Stress-CAF to Promote Chordoma Progression via Targeting ITGB1

Tao-Lan Zhang, Bo-Wen Zheng, Chao Xia, Peng-Fei Wu, Bo-Yv Zheng, Ling-Xiang Jiang, Jing Li, Guo-Hua Lv, Hong Zhou, Wei Huang* and Ming-Xiang Zou**

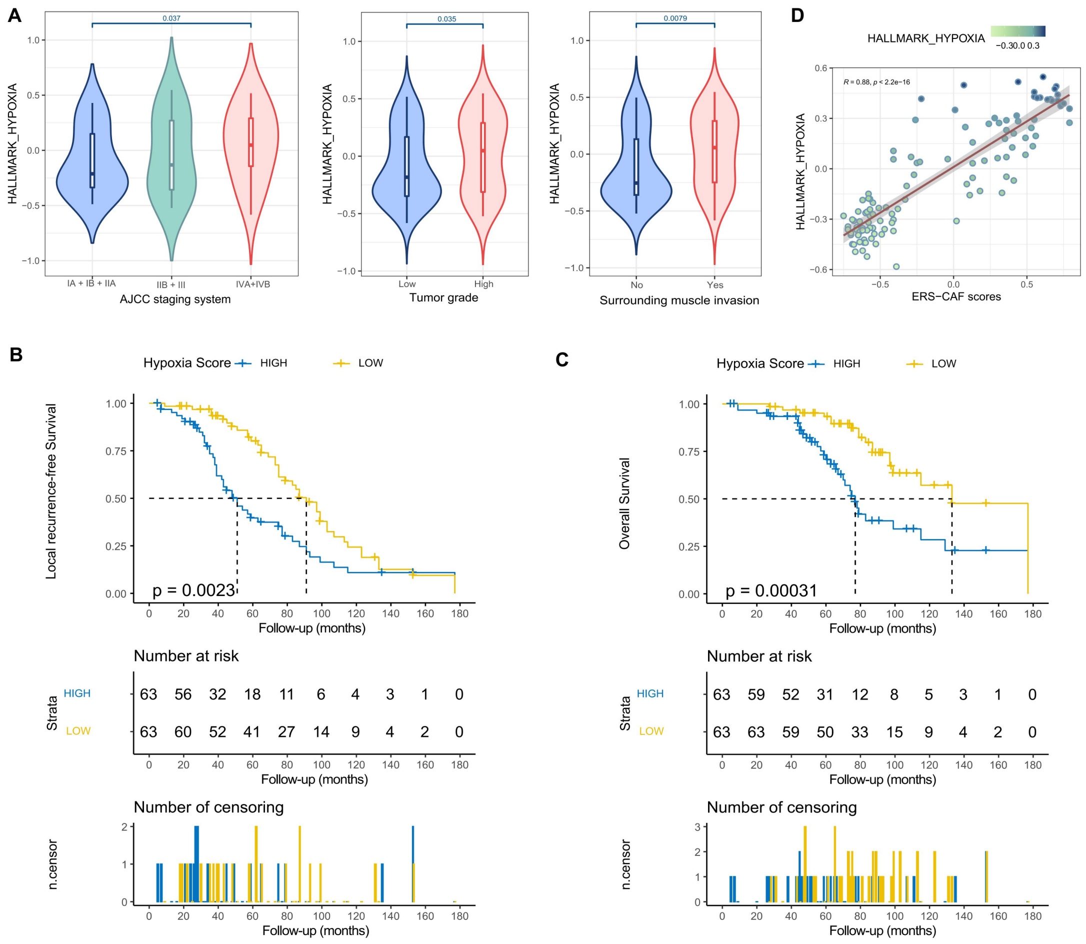

Supplementary Figure S1.

(A) The association between hypoxic signature score and AJCC staging system, tumor grade, and surrounding muscle invasion of 126 chordoma patients. (B) Kaplan–Meier curves of local recurrence-free survival (LRFS) of 126 chordoma patients categorized by hypoxic signature score. (C) Kaplan–Meier curves of overall survival (OS) of 126 chordoma patients categorized by hypoxic signature score. (D) The association between hypoxic signature score and ERS-CAF score.

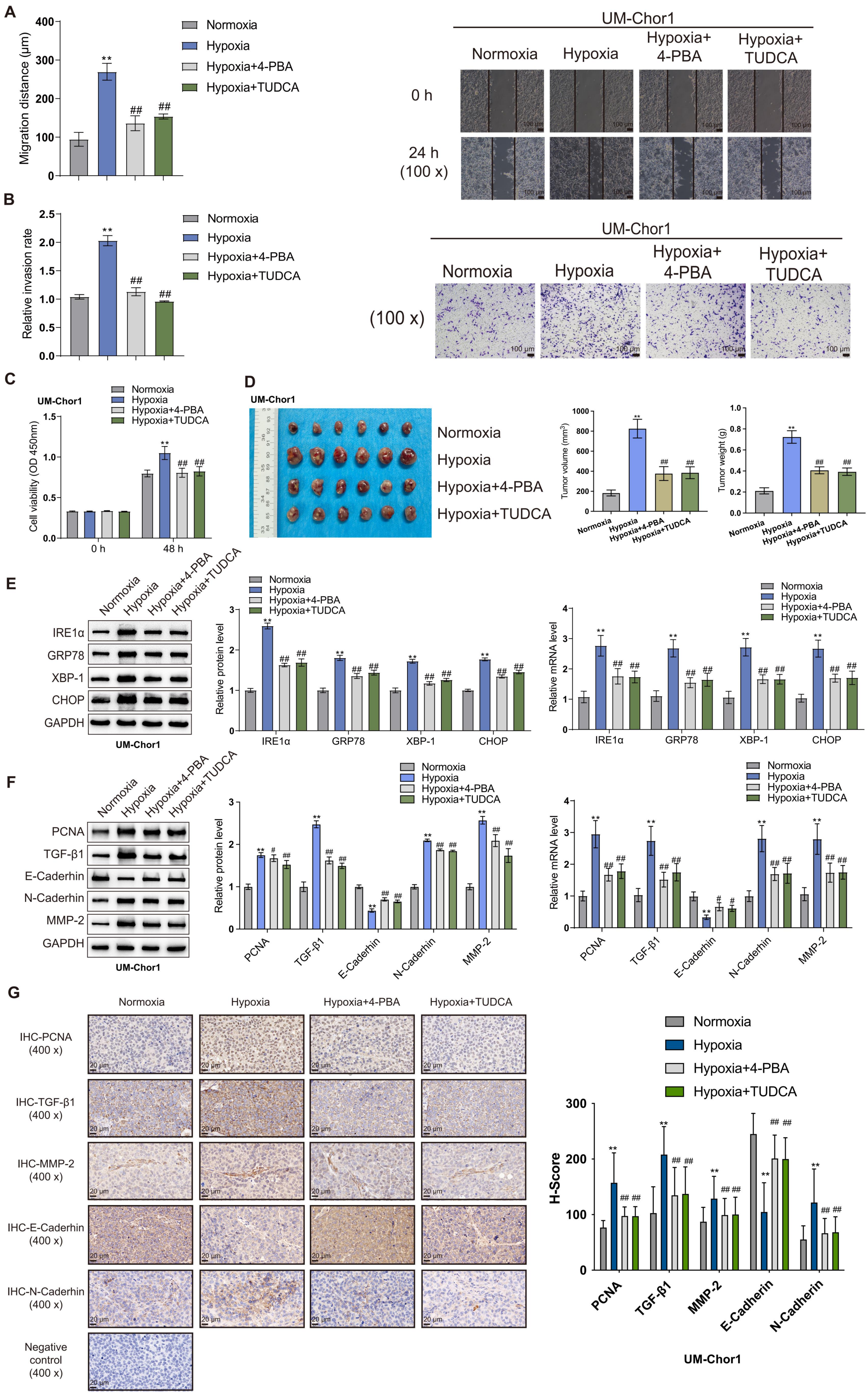

Supplementary Figure S2.

(A) Wound healing assay shows enhanced migration ability of UM-Chor1 chordoma cells co-cultured with endoplasmic reticulum stress related CAF (ERS-CAF), while blocking ERS response of CAF with 4-phenylbutyric acid (4-PBA) and tauroursodeoxycholic acid (TUDCA) reversed the above phenomenon. (B) Transwell assay reveals increased invasion ability of UM-Chor1 chordoma cells co-cultured with ERS-CAF, while blocking ERS response of CAF with 4-PBA and TUDCA reversed the above phenomenon. (C) Cell Counting Kit-8 (CCK8) assay demonstrates enhanced proliferation activity of UM-Chor1 chordoma cells co-cultured with ERS-CAF, while blocking ERS response of CAF with 4-PBA and TUDCA reversed the above phenomenon. (D) Macroscopic image of subcutaneous tumor formation in mice, indicating increased tumor weight and volume after co-transplantation with UM-Chor1 chordoma cells and ERS-CAF, while blocking ERS response of CAF with 4-PBA and TUDCA reversed the above phenomenon. (E) Western blot (WB) and qRT-PCR revealed ERS related genes expression was increased in subcutaneous tumor samples after co-transplantation with UM-Chor1 chordoma cells and ERS-CAF, while blocking ERS response of CAF with 4-PBA and TUDCA reversed the above phenomenon. (F) WB and qRT-PCR revealed EMT related proteins (including PCNA, TGF-B1, N-cadherin, and MMP-2) expression were increased in subcutaneous tumor samples after co-transplantation with UM-Chor1 chordoma cells and ERS-CAF, while blocking ERS response of CAF with 4-PBA and TUDCA reversed the above phenomenon. (G) Immunohistochemical analysis of tumor samples showed high expression of EMT-related genes after co-transplantation with UM-Chor1 chordoma cells and ERS-CAF, while blocking ERS response of CAF with 4-PBA and TUDCA reversed the above phenomenon.

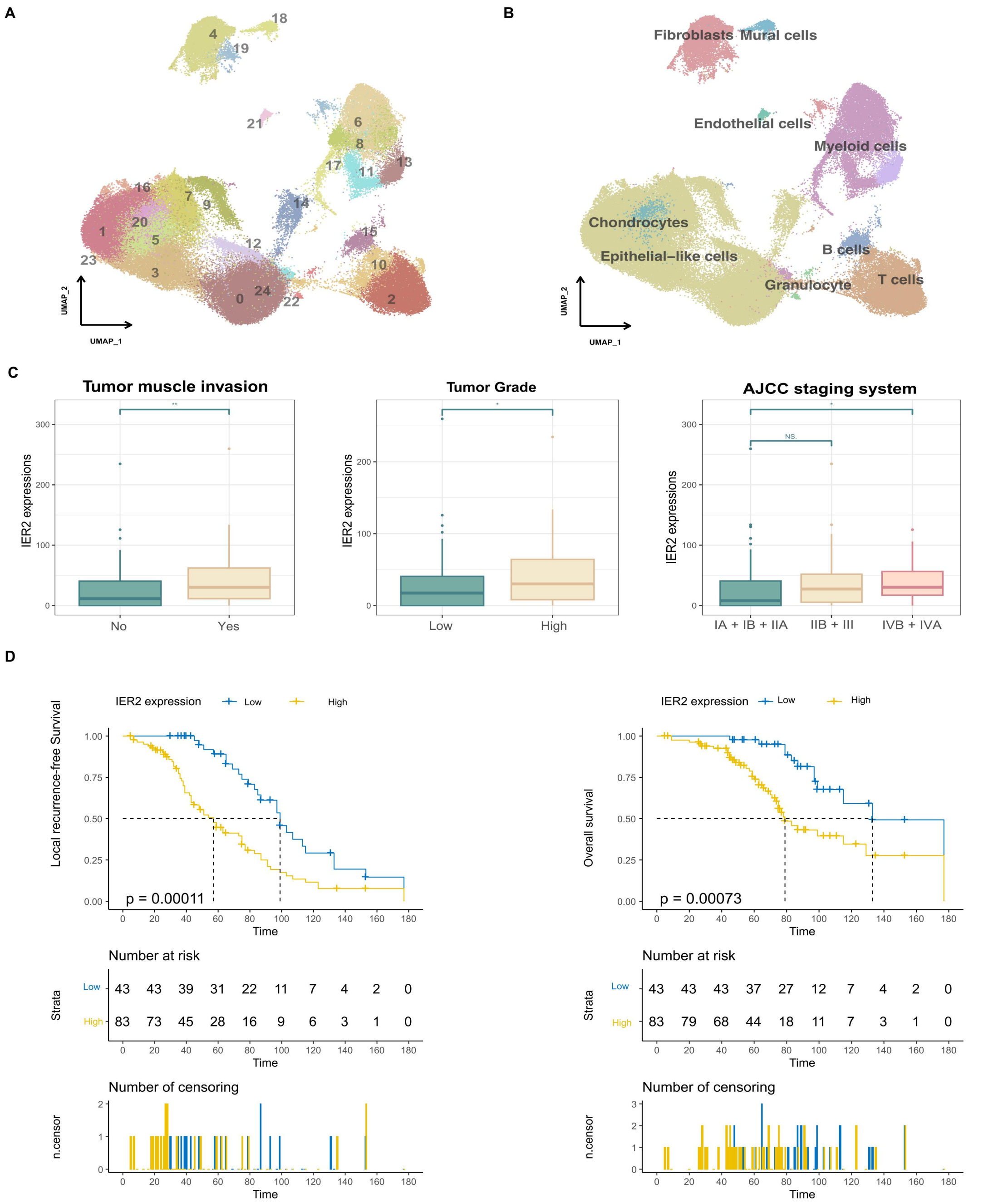

Supplementary Figure S3.

(A) UMAP showcasing 25 cellular subgroups recognized from the scRNA-seq dataset. (B) UMAP diagram of cell types corresponding to each cluster. (C) The association between IER2 expression and AJCC staging system, tumor grade, and surrounding muscle invasion of 126 chordoma patients. (D) Kaplan–Meier curves of local recurrence-free survival and overall survival of 126 chordoma patients categorized by IER2 expression.

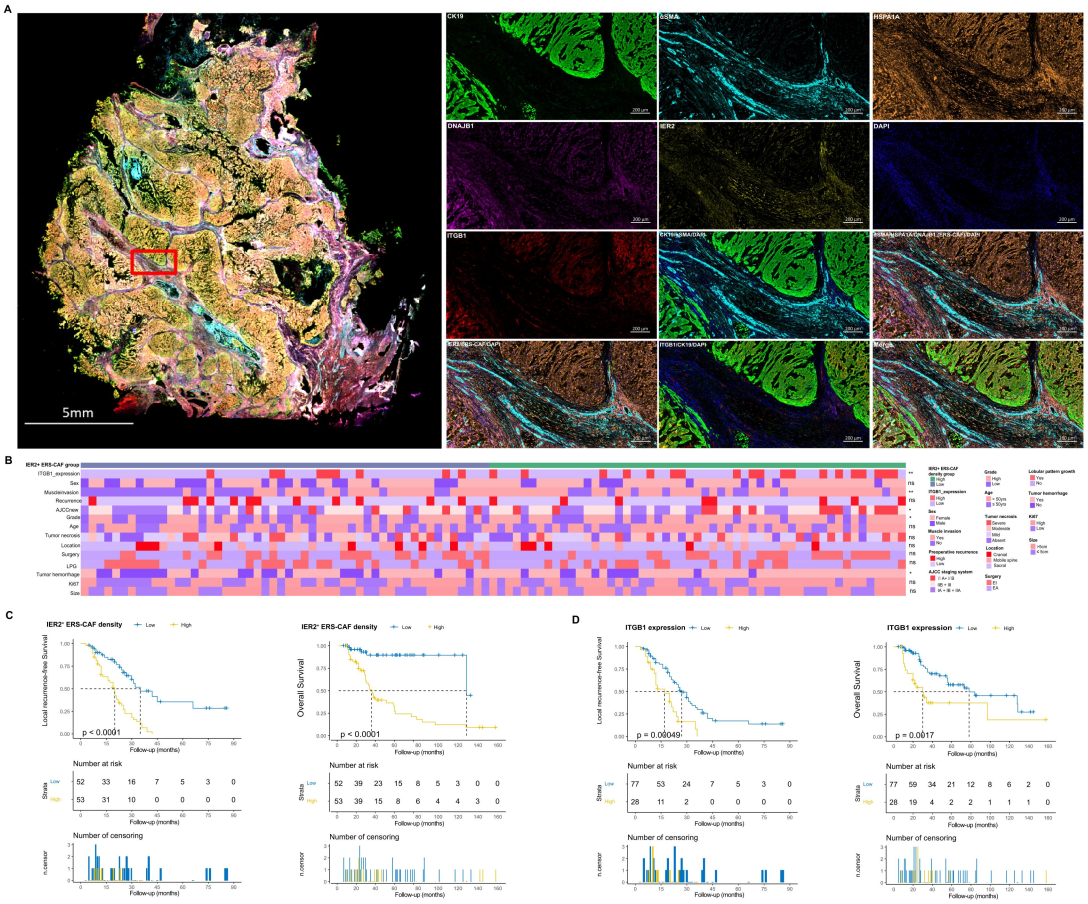

Supplementary Figure S4.

(A) Representative immunofluorescence images showing expression of QIF markers in chordoma tissues (specifically DAPI for all cells, CK19 for tumor cells, αSMA for CAFs, DNAJB1 and HSPA1A for ERS-CAF). (B) The heatmap shows the distribution of clinicopathological characteristics, IER2^{+} ERS-CAF density. (C) Kaplan–Meier curves of LRFS and OS of 105 chordoma patients stratified by IER2^{+} ERS-CAF density. (D) Kaplan–Meier curves of LRFS and OS of 105 chordoma patients stratified by ITGB1 expression.

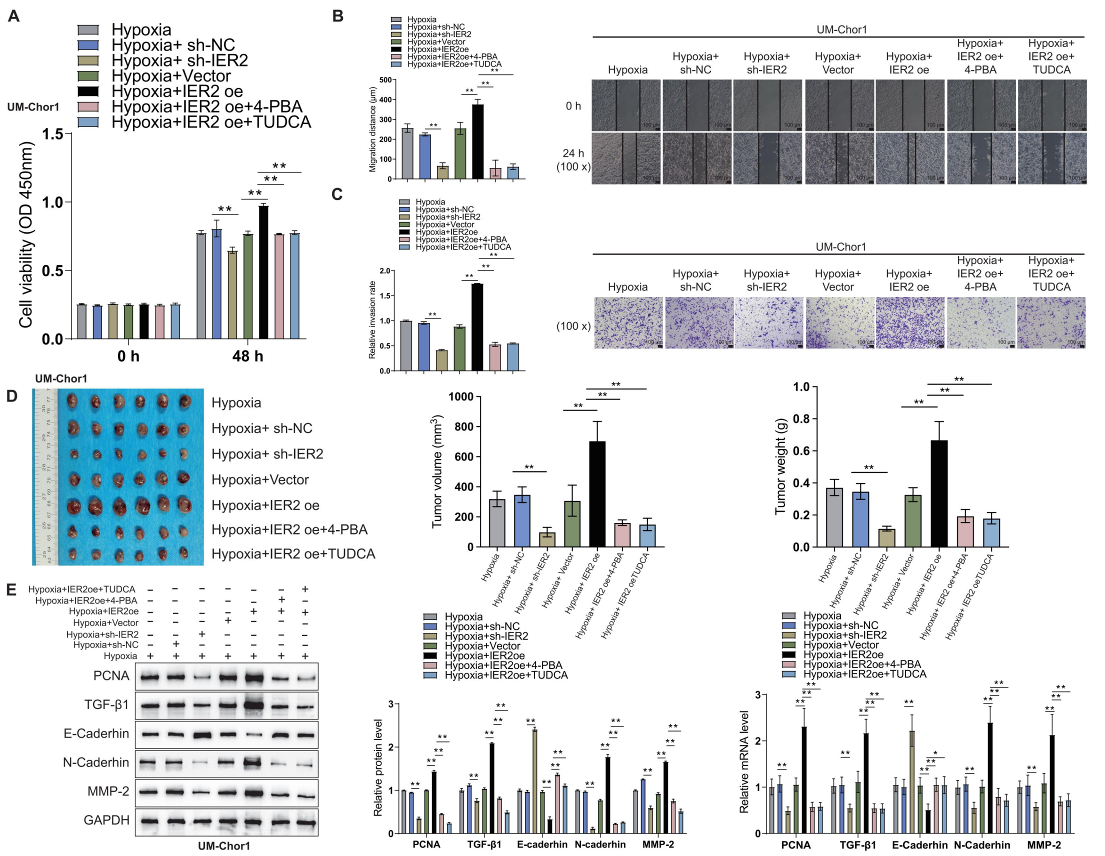

Supplementary Figure S5.

(A) CCK8 assay demonstrates enhanced proliferation activity of UM-Chor1 chordoma cells co-cultured with CAFs having IER2 overexpression, while genetic knockdown of IER2 in CAF and administering with anti-ERS agents reversed the above phenomenon. (B) Wound healing assay shows enhanced migration ability of UM-Chor1 chordoma cells co-cultured with CAFs having IER2 overexpression, while genetic knockdown of IER2 in CAF and administering with anti-ERS agents reversed the above phenomenon. (C) Transwell assay reveals increased invasion ability of UM-Chor1 chordoma cells co-cultured with CAFs having IER2 overexpression, while genetic knockdown of IER2 in CAF and administering with anti-ERS agents reversed the above phenomenon. (D) Macroscopic image of subcutaneous tumor formation in mice, indicating increased tumor weight and volume after co-transplantation with UM-Chor1 chordoma cells and CAFs having IER2 overexpression, while genetic knockdown of IER2 in CAF and administering with anti-ERS agents reversed the above phenomenon. (E) WB and qRT-PCR revealed EMT related proteins expression were increased in subcutaneous tumor samples after co-transplantation with UM-Chor1 chordoma cells and CAFs having IER2 overexpression, while genetic knockdown of IER2 in CAF and administering with anti-ERS agents reversed the above phenomenon.

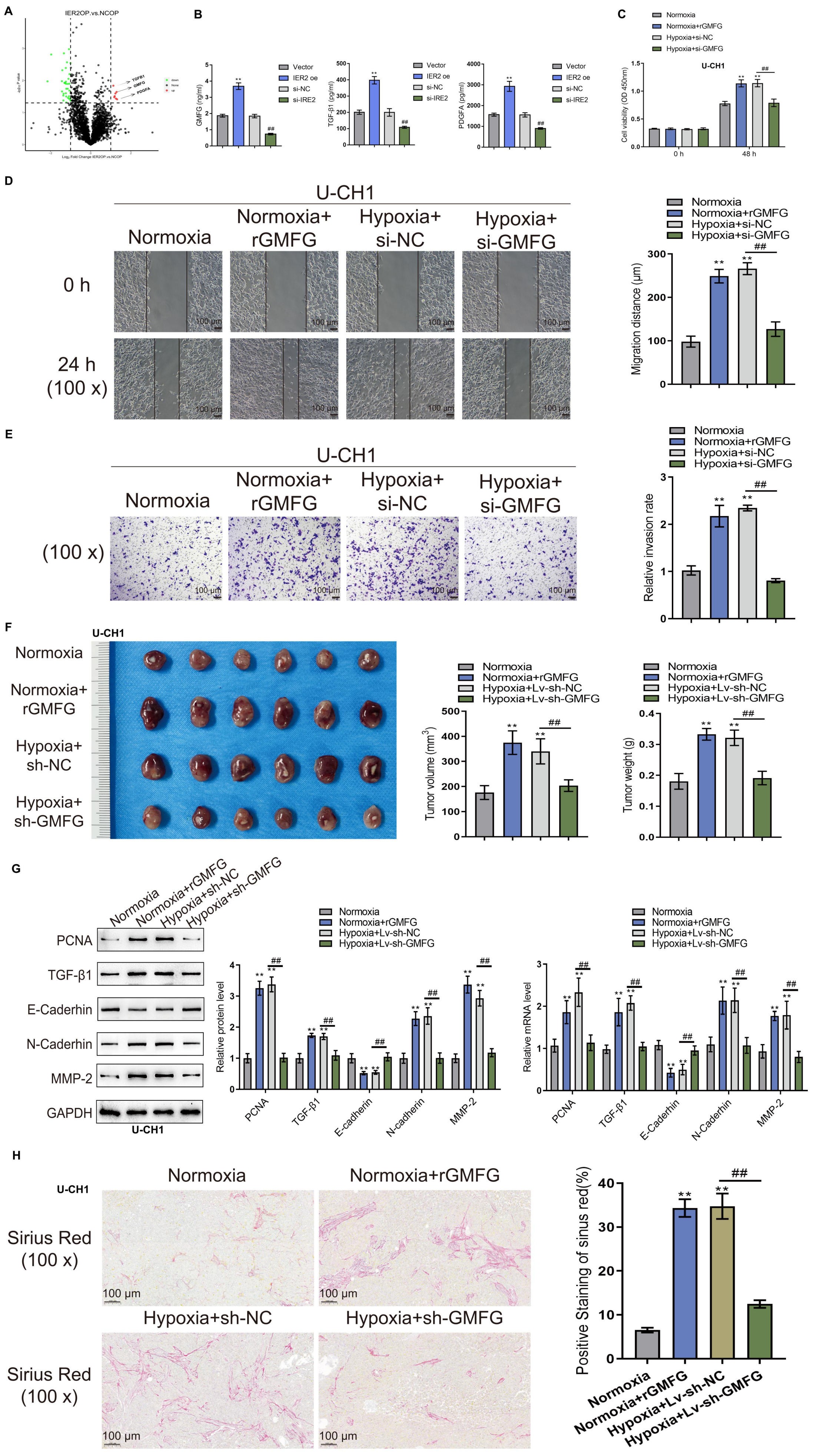

Supplementary Figure S6.

(A) Proteomic differential expression analysis of CAF supernatants before and after IER2 knockout showed that GMFG was the most significantly changed secreted protein. (B) ELISA assay showed that overexpression of IER2 significantly increased the abundance of GMFG in CAF supernatants, and IER2 knockdown significantly reduced GMFG levels in CAF supernatants. (C) CCK8 assay demonstrates enhanced proliferation activity of U-CH1 chordoma cells when introducing exogenous GMFG to CAF culture, while GMFG silence on CAF reversed the above phenomenon. (D) Wound healing assay shows enhanced migration ability of U-CH1 chordoma cells when introducing exogenous GMFG to CAF culture, while GMFG silence on CAF reversed the above phenomenon. (E) Transwell assay reveals increased invasion ability of U-CH1 chordoma cells when introducing exogenous GMFG to CAF culture, while GMFG silence on CAF reversed the above phenomenon. (F) Macroscopic image of subcutaneous tumor formation in mice, indicating increased tumor weight and volume after co-transplantation with U-CH1 chordoma cells and CAFs introducing exogenous GMFG, while GMFG silence on CAF reversed the above phenomenon. (G) WB and qRT-PCR revealed EMT related proteins expression were increased in subcutaneous tumor samples after co-transplantation with U-CH1 chordoma cells and CAFs introducing exogenous GMFG, while GMFG silence on CAF reversed the above phenomenon. (H) Trichrome and Picrosirius Red showed that after co-transplantation with U-CH1 chordoma cells and CAFs introducing exogenous GMFG, the content of type I collagen and collagen fibers in the tumor was higher, while GMFG silence on CAF reversed the above phenomenon.

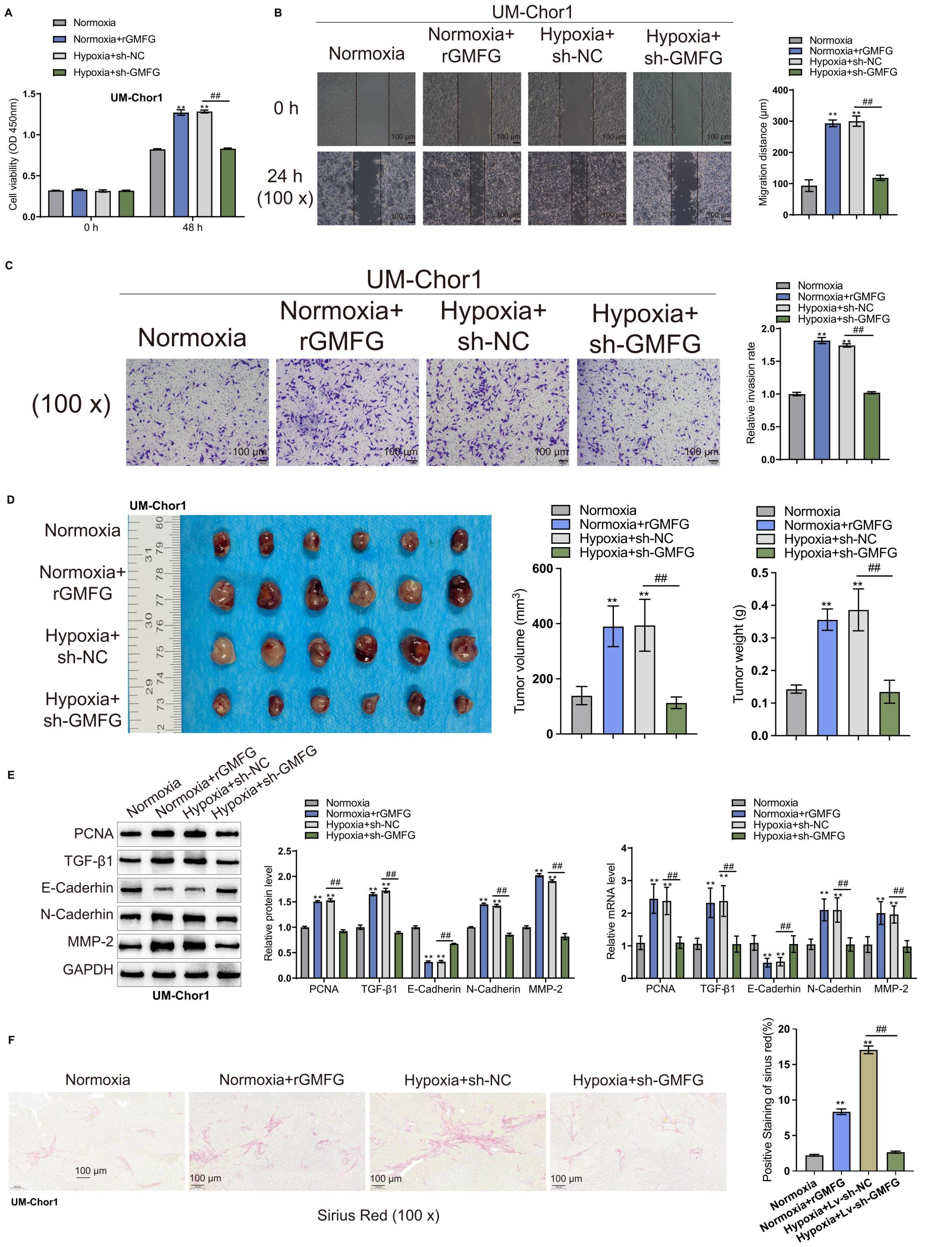

Supplementary Figure S7.

(A) CCK8 assay demonstrates enhanced proliferation activity of UM-Chor1 chordoma cells when introducing exogenous GMFG to CAF culture, while GMFG silence on CAF reversed the above phenomenon. (B) Wound healing assay shows enhanced migration ability of UM-Chor1 chordoma cells when introducing exogenous GMFG to CAF culture, while GMFG silence on CAF reversed the above phenomenon. (C) Transwell assay reveals increased invasion ability of UM-Chor1 chordoma cells when introducing exogenous GMFG to CAF culture, while GMFG silence on CAF reversed the above phenomenon. (D) Macroscopic image of subcutaneous tumor formation in mice, indicating increased tumor weight and volume after co-transplantation with UM-Chor1 chordoma cells and CAFs introducing exogenous GMFG, while GMFG silence on CAF reversed the above phenomenon. (E) WB and qRT-PCR revealed EMT related proteins expression were increased in subcutaneous tumor samples after co-transplantation with UM-Chor1 chordoma cells and CAFs introducing exogenous GMFG, while GMFG silence on CAF reversed the above phenomenon. (F) Trichrome and Picrosirius Red showed that after co-transplantation with UM-Chor1 chordoma cells and CAFs introducing exogenous GMFG, the content of type I collagen and collagen fibers in the tumor was higher, while GMFG silence on CAF reversed the above phenomenon.

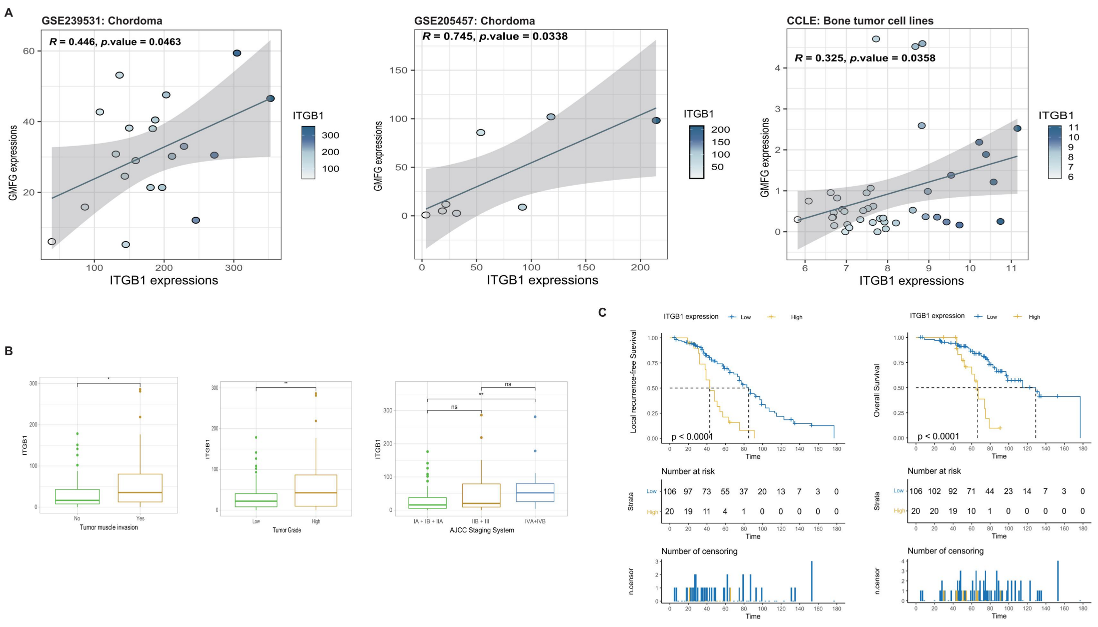

Supplementary Figure S8.

(A) Analysis of two chordoma bulk RNA-seq data sets in the GEO database and RNA-seq data of 42 bone tumor cell lines in the Cancer Cell Line Encyclopedia (CCLE) database showed a significant correlation between ITGB1 and GMFG. (B) The association between ITGB1 expression and AJCC staging system, tumor grade, and surrounding muscle invasion of 126 chordoma patients. (C) Kaplan–Meier curves of LRFS and OS of 126 chordoma patients stratified by ITGB1 expression.

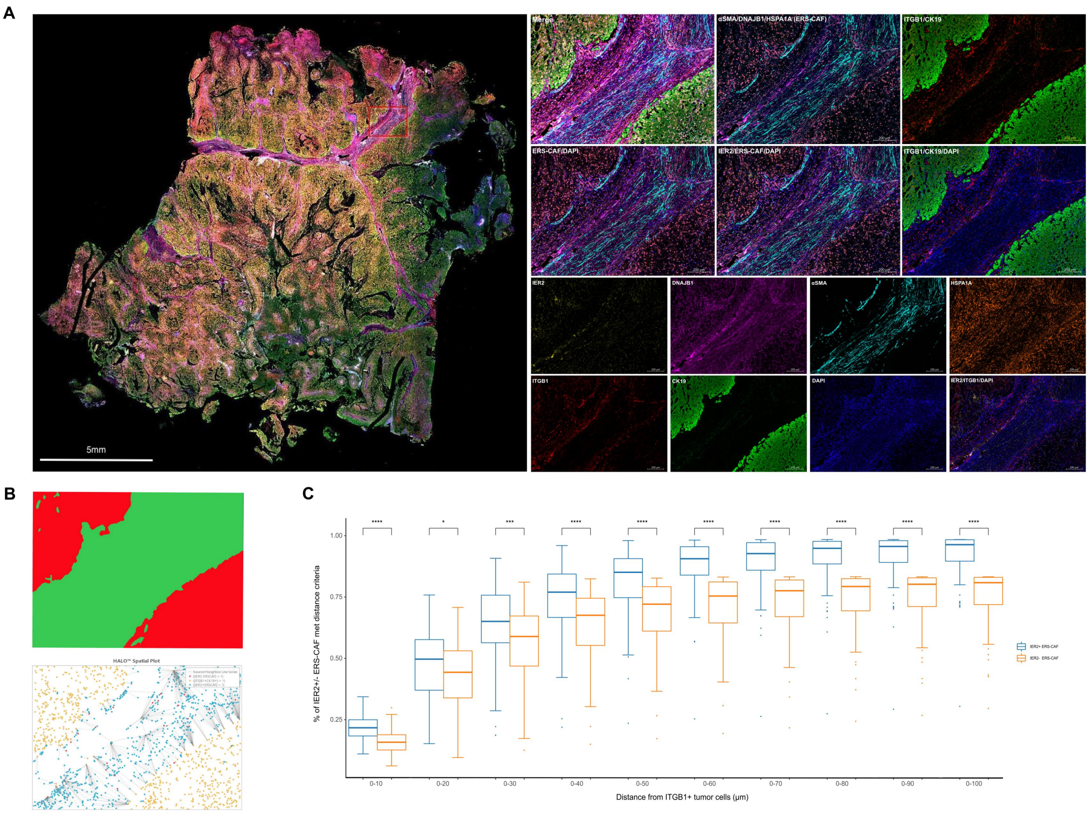

Supplementary Figure S9.

(A) Representative immunofluorescence images showing expression of QIF markers in chordoma tissues (specifically DAPI for all cells, CK19 for tumor cells, αSMA for CAFs, DNAJB1 and HSPA1A for ERS-CAF). (B) Proximity analysis of $IER2^{+}$ ERS-CAF to $ITGB1^{+}$ tumor cells. (C) The effective percentage of $IER2^{+}$ ERS-CAF and $IER2^{-}$ ERS-CAF within a range between 0 and 100 μm radii of $ITGB1^{+}$ tumor cells in consecutively increasing 10 μm steps.

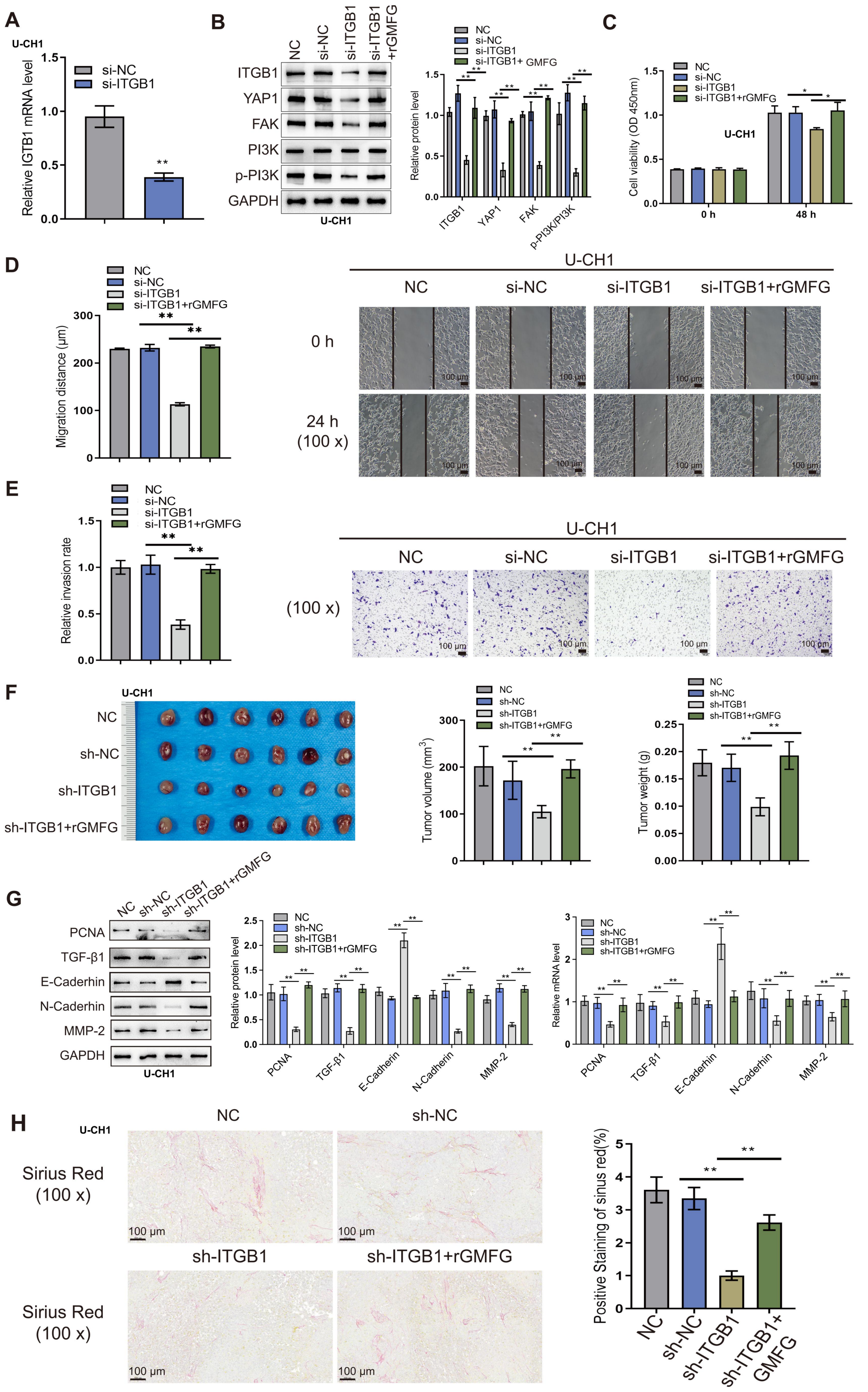

Supplementary Figure S10.

(A) qRT-PCR revealed showed U-CH1 cell line featuring successful ITGB1 knockdown. (B) WB and qRT-PCR revealed exogenous GMFG promoted the expression of ITGB1 and its downstream molecules (including YAP1, FAK, PI3K and p-PI3K) on tumor cells. (C) CCK8 assay demonstrates attenuated proliferation activity of U-CH1 chordoma cells featuring ITGB1 knockdown, while exogenous GMFG addition reversed the above phenomenon. (D) Wound healing assay shows attenuated migration ability of U-CH1 chordoma cells featuring ITGB1 knockdown, while exogenous GMFG addition reversed the above phenomenon. (E) Transwell assay reveals attenuated invasion ability of U-CH1 chordoma cells featuring ITGB1 knockdown, while exogenous GMFG addition reversed the above phenomenon. (F) Macroscopic image of subcutaneous tumor formation in mice, indicating decreased tumor weight and volume after co-transplantation with CAFs and U-CH1 chordoma cells featuring ITGB1 knockdown, while exogenous GMFG addition reversed the above phenomenon. (G) WB and qRT-PCR revealed EMT related proteins expression were decreased in subcutaneous tumor samples after co-transplantation with CAFs and U-CH1 chordoma cells featuring ITGB1 knockdown, while exogenous GMFG addition reversed the above phenomenon. (H) Trichrome and Picrosirius Red showed that after co-transplantation with CAFs and U-CH1 chordoma cells featuring ITGB1 knockdown, the content of type I collagen and collagen fibers in the tumor was lower, while exogenous GMFG addition reversed the above phenomenon.

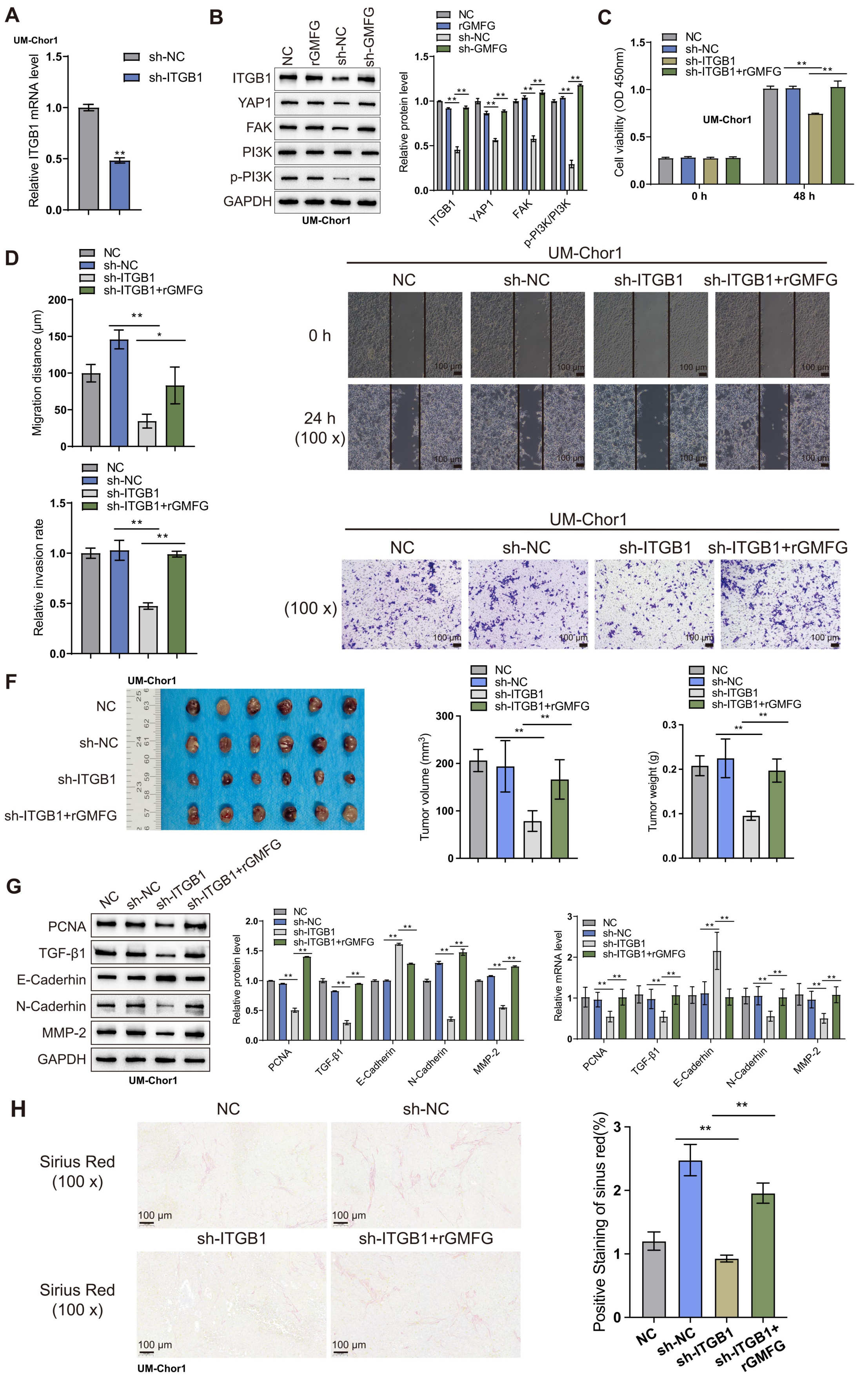

Supplementary Figure S11.

(A) qRT-PCR revealed showed UM-Chor1 cell line featuring successful ITGB1 knockdown. (B) WB and qRT-PCR revealed exogenous GMFG promoted the expression of ITGB1 and its downstream molecules (including YAP1, FAK, PI3K and p-PI3K) on tumor cells. (C) CCK8 assay demonstrates attenuated proliferation activity of UM-Chor1 chordoma cells featuring ITGB1 knockdown, while exogenous GMFG addition reversed the above phenomenon. (D) Wound healing assay shows attenuated migration ability of UM-Chor1 chordoma cells featuring ITGB1 knockdown, while exogenous GMFG addition reversed the above phenomenon. (E) Transwell assay reveals attenuated invasion ability of UM-Chor1 chordoma cells featuring ITGB1 knockdown, while exogenous GMFG addition reversed the above phenomenon. (F) Macroscopic image of subcutaneous tumor formation in mice, indicating decreased tumor weight and volume after co-transplantation with CAFs and UM-Chor1 chordoma cells featuring ITGB1 knockdown, while exogenous GMFG addition reversed the above phenomenon. (G) WB and qRT-PCR revealed EMT related proteins expression were decreased in subcutaneous tumor samples after co-transplantation with CAFs and UM-Chor1 chordoma cells featuring ITGB1 knockdown, while exogenous GMFG addition reversed the above phenomenon. (H) Trichrome and Picrosirius Red showed that after co-transplantation with CAFs and UM-Chor1 chordoma cells featuring ITGB1 knockdown, the content of type I collagen and collagen fibers in the tumor was lower, while exogenous GMFG addition reversed the above phenomenon.

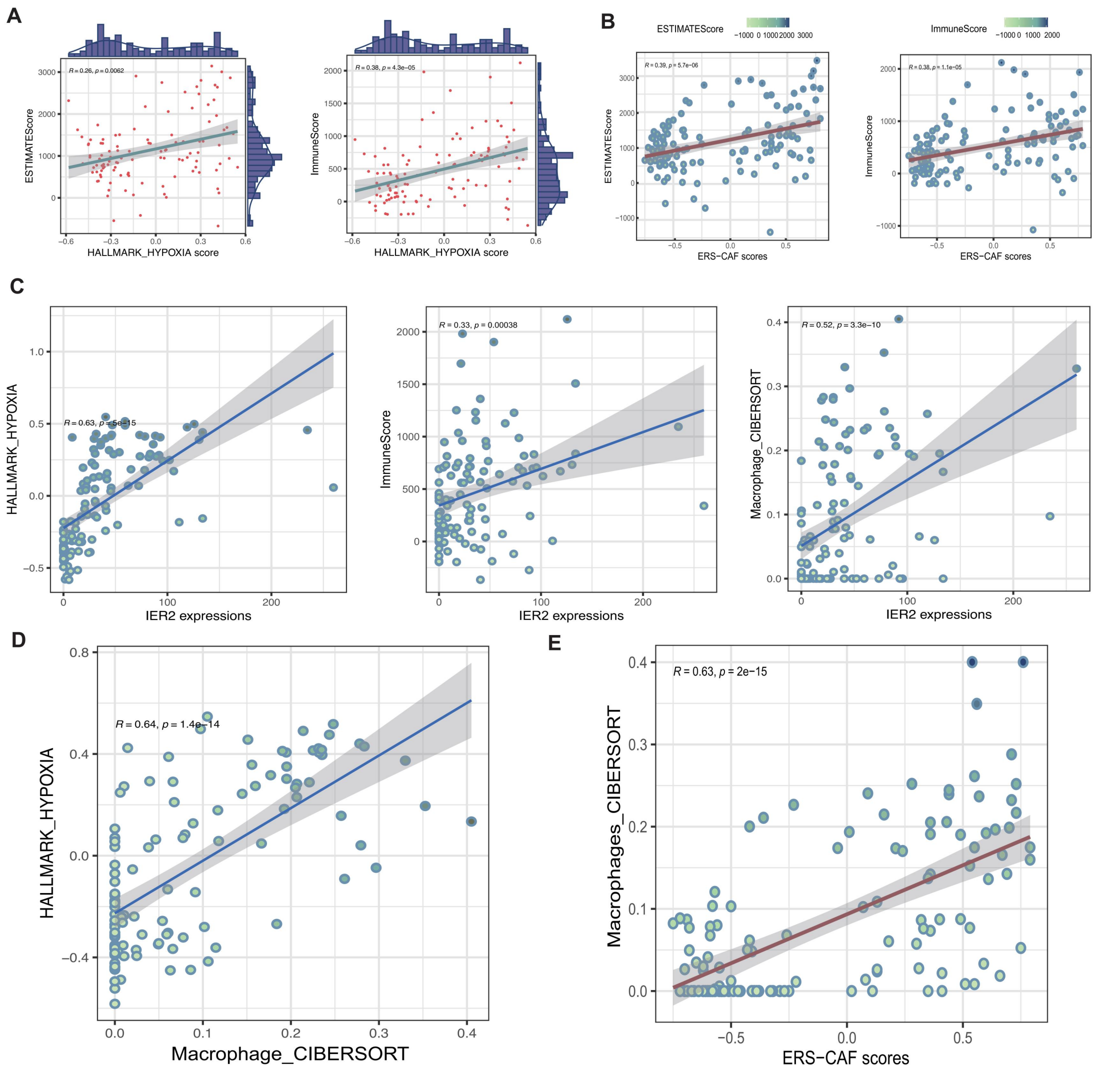

Supplementary Figure S12.

(A) The association between HALLMARK_HYPOXIA score and ESTIMATEScore and ImmuneScore of 126 chordoma patients. (B) The association between ERS-CAF score and ESTIMATEScore and ImmuneScore of 126 chordoma patients. (C) The association between IER2 expession and ESTIMATEScore, ImmuneScore, and Macrophage_CIBERSORT score of 126 chordoma patients. (D) The association between Macrophage_CIBERSORT score and ESTIMATEScore of 126 chordoma patients. (E) The association between Macrophage_CIBERSORT score and ERS-CAF Score of 126 chordoma patients.

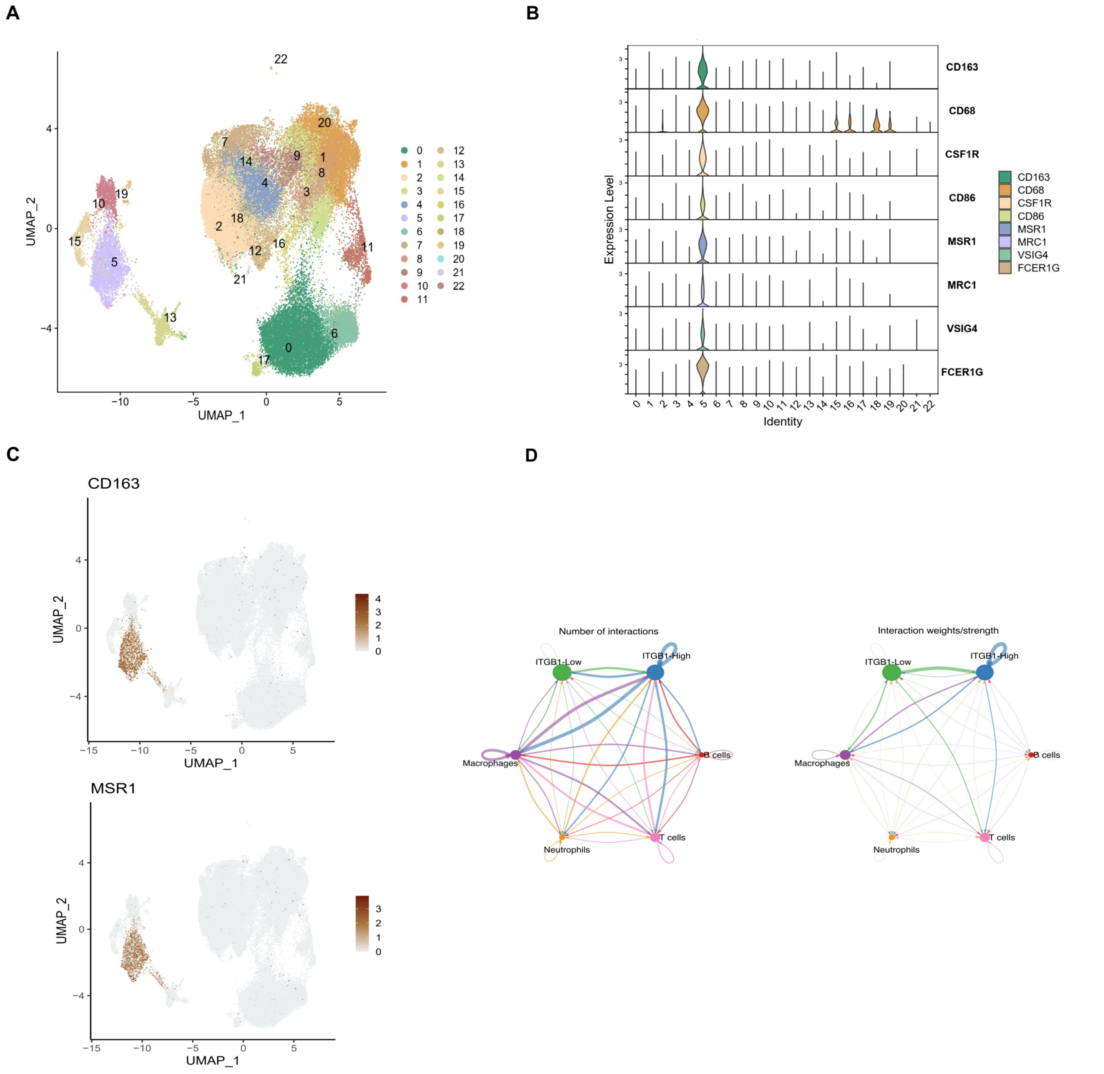

Supplementary Figure S13.

(A) UMAP projection of 23 myeloid cells clusters across all samples. (B) Violin plot showing the expression levels of macrophages-related DEGs in 23 clusters, indicating that cluster 5 represents macrophages. (C) UMAP representation of macrophage-specific markers. (D) Circular plot illustrating the quantity of interactions and interaction weight/strength among diverse cell types, and the line thickness is proportional to the number of ligands-receptors pairs. There is a significantly strong interaction between ITGB1^{high} tumor cells and macrophages.

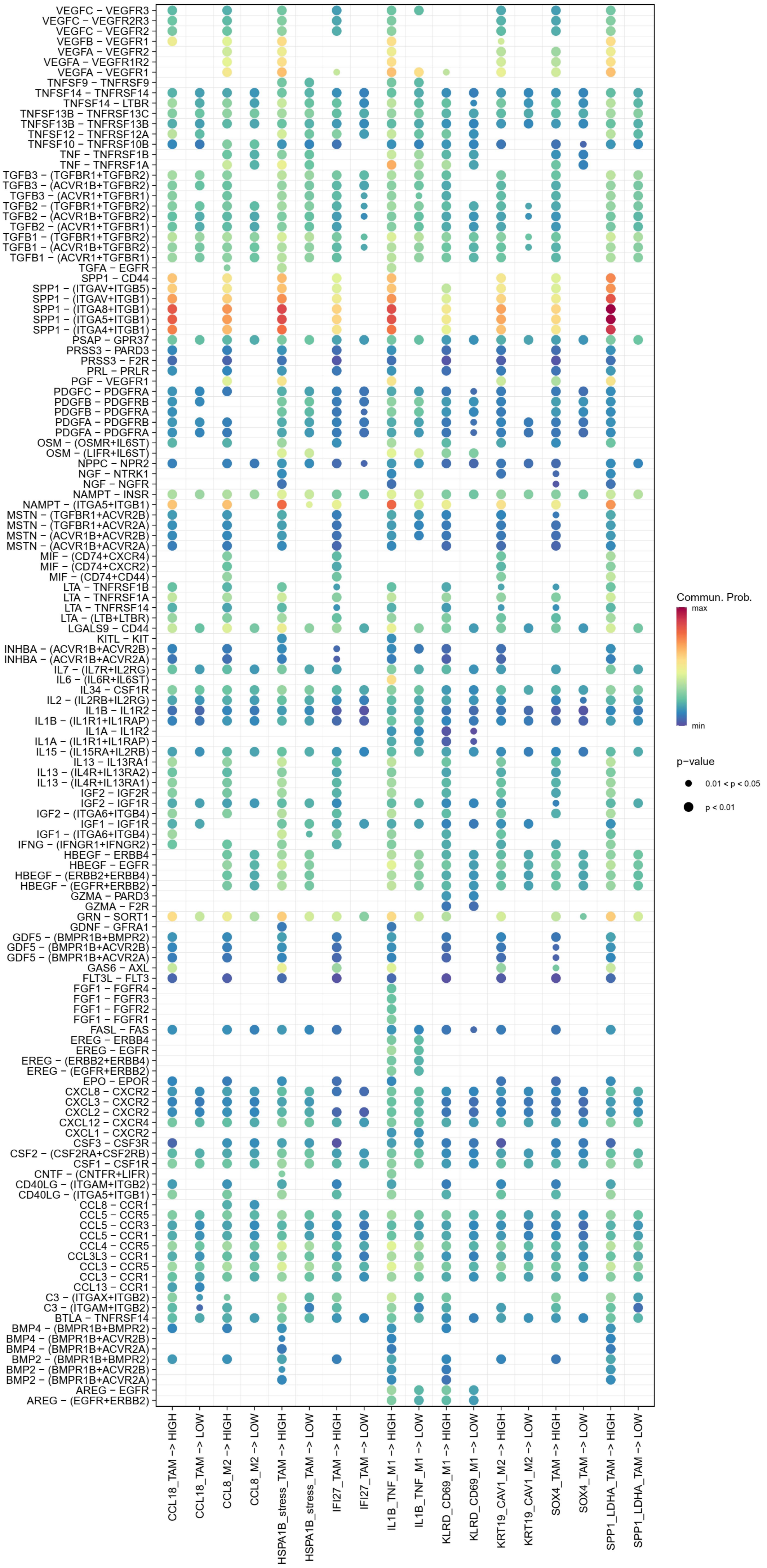

Supplementary Figure S14.

Cellchat results revealed the strongest interaction between SPP1^{+} tumor-associated macrophages and ITGB1^{high} tumor cells.

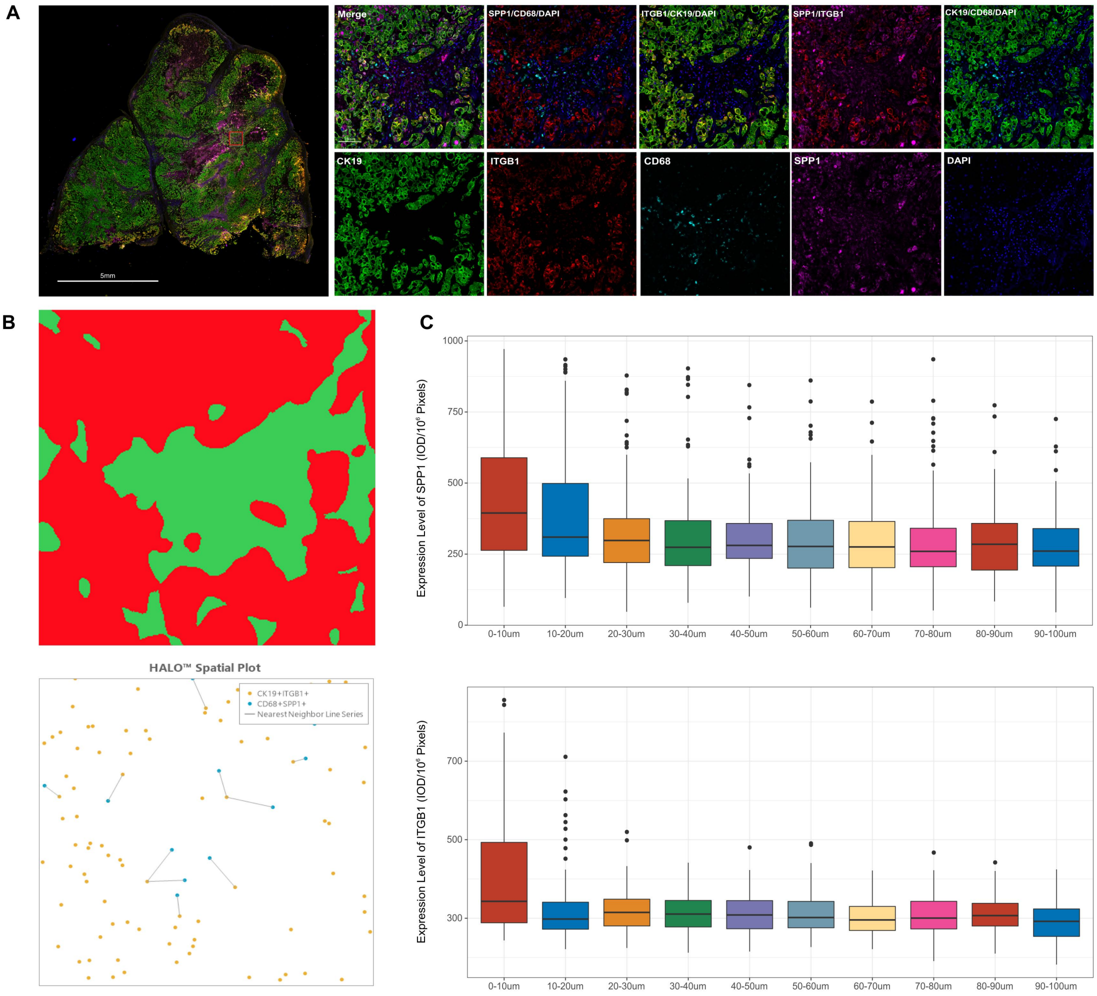

Supplementary Figure S15.

(A) Representative immunofluorescence images showing expression of QIF markers in chordoma tissues (specifically DAPI for all cells, CK19 for tumor cells, αSMA for CAFs, CD68 for tumor-associated macrophage). (B) Proximity analysis of SPP1^{+} macrophage to ITGB1^{+} tumor cells. (C) Differential expression of SPP1 (up) /ITGB1 (down) signaling according to the distance between tumor-associated macrophage and tumor cells. The differences in expression of SPP1/ITGB1 between each group and the 0-10um group were analyzed.
